# Review: Trace and Residual Rare-Earth Effects on Inclusion Evolution and Nb-Ti-V Precipitation in Microalloyed Steels

**DOI:** 10.3390/ma19132768

**Published:** 2026-06-30

**Authors:** Guomin Wei, Minghe Li, Bo Cui, Hongrui Li, Asmawan Mohd Sarman

**Affiliations:** 1 School of Mechanical and Civil Engineering, Jilin Agricultural Science and Technology College, Jilin 132101, China; weiguomin@jlnku.edu.cn (G.W.);; 2 Civil Engineering, Faculty of Engineering, University Malaysia Sabah, Jalan UMS, Kota Kinabalu 88400, Sabah, Malaysia

**Keywords:** trace rare earth elements, inclusion evolution, NbC/TiN precipitation, microalloyed steels, rare earth inclusions

## Abstract

This review focuses on the effects of trace and residual rare-earth elements on inclusion evolution and Nb–Ti–V precipitation behavior in microalloyed steels. Existing studies indicate that trace rare-earth elements can transform conventional Al_2_O_3_- and MnS-type inclusions into rare-earth oxides, oxysulfides, and sulfides, while also modifying local interfacial states and solute distributions through segregation and interfacial activity. These changes further affect the nucleation sites, growth behavior, coarsening tendency, and spatial distribution of NbC, TiN, VC, and related carbonitrides. To explain the seemingly contradictory precipitation responses reported in the literature, this review examines rare-earth effects from the perspectives of inclusion inheritance, heterogeneous nucleation, interfacial energy modification, local solute redistribution, and thermomechanical processing history. The available evidence suggests that the metallurgical role of trace rare-earth elements cannot be attributed solely to inclusion modification. Instead, their effects arise from the combined influence of inclusion evolution, interfacial activity, local chemical heterogeneity, and precipitation kinetics under specific processing conditions. These insights provide practical guidance for alloy and process design by linking rare-earth addition, inclusion control, and Nb–Ti–V precipitation regulation in microalloyed steels.

## 1. Introduction

Due to their combination of high strength, good toughness, weldability, and formability, microalloyed steels are widely used in structural engineering, pipelines, automotive manufacturing, and marine engineering. Their properties depend not only on the matrix microstructure but also on inclusion evolution and the precipitation behavior of microalloying elements [1,2]. The former affects steel cleanliness, solidification morphology, microsegregation, and crack initiation, whereas the latter mainly involves the nucleation, growth, coarsening, dissolution, and spatial distribution of carbides, nitrides, and carbonitrides formed by microalloying elements such as Nb, Ti, and V. These precipitates play a critical role in precipitation strengthening, grain-boundary pinning, and microstructural stability [3].

Rare-earth elements, particularly La and Ce, have long been recognized as effective inclusion modifiers in steels. Previous studies have shown that rare earths can promote the transformation of Al_2_O_3_- or MnS-based inclusions into smaller, more rounded, and more uniformly distributed rare-earth oxides, oxysulfides, and sulfides, thereby markedly altering inclusion formation and inheritance behavior [4,5]. Recent studies have further revealed that rare-earth elements not only modify inclusions themselves but also influence the nucleation, growth, and spatial distribution of microalloy precipitates such as NbC, TiN, and (Nb,Ti)(C,N) [2,6,7]. Therefore, in microalloyed steels, the metallurgical effects of rare-earth elements are not limited to inclusion modification during steelmaking, but extend to inclusion evolution, interfacial reconstruction, precipitation nucleation, and subsequent microstructural development.

Although research on rare-earth-containing steels has become increasingly extensive, the current understanding remains relatively fragmented. Existing reviews have mainly focused on rare-earth-induced grain refinement during solidification, improvements in oxide metallurgy and weldability, and the mechanisms of second-phase-induced heterogeneous nucleation and microstructural refinement [8,9]. A growing number of studies have shown that even at ppm-level concentrations, the metallurgical effects of rare-earth elements cannot be ignored [3,5,10,11,12]. However, systematic understanding remains limited regarding how trace and residual rare-earth elements influence inclusion evolution in microalloyed steels and further regulate microalloy precipitation behavior. Trace or residual rare-earth elements may significantly affect microstructural evolution through interfacial segregation, inclusion transformation, and local solute redistribution. In this review, “trace and residual rare earths” are used in a practical metallurgical sense to describe rare-earth elements retained in steel after deoxidation, desulfurization, and solidification reactions. Previous studies have reported that residual rare earths originating from rare-earth-bearing raw materials may be present at only a few ppm levels, for example, 3–8 ppm La, while still producing significant metallurgical effects [13]. Therefore, this term does not imply a strict compositional classification, but refers broadly to rare-earth elements present from several ppm to low hundred-ppm levels. For brevity, these elements are collectively referred to as “trace rare earths” in the following sections.

It is worth noting that the reported effects of rare-earth elements on precipitation behavior remain inconsistent. Some studies suggest that La may increase the solubility of Nb and C in the austenitic region, thereby delaying strain-induced NbC precipitation [6], whereas others have found that La may promote NbC precipitation in bcc Fe [7]. In addition, in certain steel grades, rare-earth inclusions can act as heterogeneous nucleation sites for TiN, MnS, or Nb-enriched carbonitrides [2,3,5], whereas in other systems, rare earths suppress the formation of coarse primary TiN, Nb-rich carbonitrides, or other high-temperature precipitates and alter their preferred precipitation locations [4,12]. These discrepancies indicate that there is no universal rule governing the effect of rare-earth elements on precipitation behavior. Instead, their influence is closely associated with effective rare-earth content, the occurrence state of rare-earth elements, including solute atoms, segregated atoms, clusters, and inclusions, the composition and evolution pathway of inherited inclusions, and the subsequent thermomechanical processing history.

At the same time, increasing evidence indicates that the total rare-earth content does not directly reflect its actual metallurgical effect. Rare-earth elements can exist in steels in various forms, including solute atoms, atomic clusters, and rare-earth-containing inclusions [14,15,16,17,18,19]. Among these, rare earths in solute or interfacially segregated states may influence the nucleation and growth of microalloy precipitates by altering interfacial energy and local chemical potential [20,21,22,23]. In contrast, rare earths incorporated into inclusions mainly affect precipitation by modifying the composition, size distribution, number density, and interfacial characteristics of inherited inclusions, which subsequently determine the availability and effectiveness of heterogeneous nucleation sites for microalloy precipitates [21]. Therefore, in microalloyed steels, the mechanisms by which trace and residual rare-earth elements act can no longer be simply described as inclusion spheroidization and refinement. Instead, they should be evaluated comprehensively from multiple perspectives, including interfacial segregation, changes in interfacial energy, and precipitation behavior.

Based on this understanding, this review examines the effects of trace and residual rare-earth elements on inclusion evolution and microalloy precipitation behavior in microalloyed steels. Particular attention is paid to the occurrence states of trace and residual rare-earth elements in steels and their significance in interfacial metallurgy, rare-earth-induced inclusion modification, inheritance, and evolution pathways, and the coupling relationship between inclusion evolution and the precipitation behavior of Nb-, Ti-, and V-related carbonitrides. This review aims to provide an integrated perspective on the interactions among trace rare-earth elements, inclusion evolution, and microalloy precipitation behavior in microalloyed steels.

## 2. Trace and Residual Rare Earth as a Hidden Metallurgical Variable in Microalloyed Steels

### 2.1. Occurrence States and Localized Action Characteristics of Trace Rare Earth

It should be noted that there is currently no universally accepted quantitative criterion for distinguishing residual, trace, and intentional rare-earth additions in steels. In this review, residual rare earths refer to ppm-level rare-earth elements retained in steels from raw materials, scrap, or previous metallurgical processes. The term trace rare earths is used in a practical metallurgical sense to describe rare-earth contents ranging from several ppm to low hundred-ppm levels, within which localized effects such as interfacial segregation, inclusion modification, and local solute redistribution can still be observed [24,25,26,27,28].

In studies of microalloyed steels, rare-earth elements are typically regarded as auxiliary elements for deoxidation, desulfurization, and inclusion modification. However, recent studies have shown that the role of rare-earth elements in steels cannot be characterized simply by their addition level or total rare-earth content. Particularly at concentrations ranging from parts per million to several hundred ppm, rare-earth elements can still exert a significant influence on microstructural evolution, despite their extremely low average concentration. These effects are mainly associated with interfacial segregation, local solute redistribution, and interfacial energy regulation. Therefore, the metallurgical effect of trace and residual rare earths cannot be evaluated solely on the basis of their total concentration. Rather, it strongly depends on their occurrence state in steel, because rare earths present as solute atoms, segregated species, clusters, or inclusion constituents may affect microstructural evolution through different mechanisms.

This review strictly distinguishes between inclusions and precipitates. The term “inclusions” primarily refers to rare-earth oxides, oxysulfides, sulfides, and their composite inclusions. By contrast, “precipitation behavior” specifically refers to the precipitation kinetics of NbC, TiN, VC, and related carbonitrides, including their nucleation, growth, coarsening, and dissolution behavior. Except when citing the original literature, this review avoids using the generalized term “second-phase precipitation” in order to prevent conceptual confusion between inclusion evolution and the precipitation behavior of microalloying elements.

Previous studies have shown that rare-earth elements in steels mainly exist in two categories of states. The first category comprises rare-earth compounds, such as RE oxides, RE oxysulfides, and RE sulfides. The second category comprises matrix-retained rare earths, including solid-solution atoms, grain-boundary-segregated atoms, and nanocluster-bound rare-earth elements [10]. Chen et al. [10] pointed out that the total rare-earth content (T.RE.) does not accurately represent the effective rare-earth content that can continue to participate in microstructural evolution, because a considerable fraction of rare-earth elements is fixed in inclusions during deoxidation and desulfurization.

**Figure 1:**
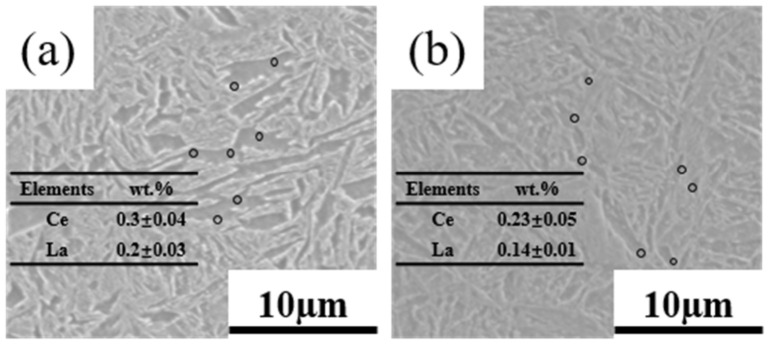
Rare-earth element segregation at grain boundaries in rare-earth microalloyed dual-phase steel [11]. (**a**) the distribution of rare earth elements Ce and La in the experimental steel annealed at 780 °C; (**b**) the distribution of rare earth elements Ce and La in the experimental steel annealed at 820 °C.

Li et al. [11] observed the local enrichment of Ce and La at grain boundaries in a rare-earth microalloyed dual-phase steel, indicating that rare-earth elements can exist not only as inclusion-forming elements but also as localized segregated species at high-energy interfacial regions. As shown in Figure 1, the enrichment of rare-earth elements along grain boundaries provides direct evidence that, at trace levels, the key factor for microstructural control is not necessarily the formation of coarse rare-earth inclusions, but rather the effective rare earth retained at grain boundaries, interfaces, and local defect regions. This local occurrence state suggests that trace rare-earth elements should be regarded as dynamic state variables that may influence interfacial stability, local diffusion, and subsequent phase transformation or precipitation behavior, rather than as simple compositional additions.

The fundamental reason why trace amounts of rare-earth elements can remain effective at low concentrations lies in the highly localized nature of their action. Unlike conventional alloying elements such as Mn and Cr, rare-earth elements exhibit pronounced interfacial activity because of their strong affinity for interfaces and defects. As a result, they preferentially segregate to grain boundaries, inclusion/matrix interfaces, precipitate/matrix interfaces, and other chemically heterogeneous regions. Such interfacial enrichment can alter local thermodynamic conditions, interfacial energies, and atomic diffusion processes, thereby affecting inclusion modification, heterogeneous nucleation, and precipitation evolution even at very low concentrations [12,29,30,31,32,33,34,35]. Although these regions occupy only a small volume fraction, they control key processes such as solidification nucleation, grain-boundary migration, microalloy precipitate nucleation, and crack initiation. Consequently, rare-earth elements at the ppm level can still produce significant microstructural effects.

Direct experimental evidence for rare-earth segregation requires spatially resolved chemical characterization at grain boundaries, interfaces, or nanoscale clusters. Atom probe tomography (APT) provides the most direct three-dimensional evidence because it can resolve atomic-scale concentration gradients and quantify rare-earth enrichment at grain boundaries, precipitate/matrix interfaces, and nanoscale clusters. Transmission electron microscopy combined with scanning transmission electron microscopy–energy-dispersive spectroscopy (TEM/STEM–EDS) can also provide direct evidence when elemental maps or line scans reveal localized RE enrichment across grain boundaries, inclusion/matrix interfaces, or precipitate/matrix interfaces. Aberration-corrected STEM–EDS or STEM–EELS is particularly useful for identifying nanoscale RE-enriched layers or clusters. In addition, Auger electron spectroscopy (AES) or secondary ion mass spectrometry (SIMS) performed on exposed grain-boundary fracture surfaces can provide complementary evidence of interfacial enrichment, although their spatial resolution and quantification accuracy are generally less suitable than those of APT for nanoscale segregation in steels.

Song and Sun [30] found that Ce addition to SA508-III steel significantly mitigated the deterioration of thermoplasticity caused by Sn. This effect was attributed to Ce-related grain-boundary segregation and the resulting enhancement of grain-boundary bonding strength, rather than simply to changes in the number of inclusions. Guo et al. [31] reported similar results in SA508-4N steel, indicating that trace amounts of rare-earth elements can improve grain-boundary stability through grain-boundary segregation. Xin et al. [29] further pointed out that Ce can form Ce–S–As or CeAs-type inclusions with As and S, thereby reducing As segregation at grain boundaries. This suggests that rare-earth elements can also alter the local chemical environment by redistributing residual elements.

In addition to grain-boundary segregation, rare-earth elements may also participate in dislocation and diffusion processes in the form of atomic clusters. Liu et al. [12] observed RE clusters in TRIP steel and proposed that they can influence dislocation motion through a solute-drag effect, while simultaneously increasing the solubility of V in austenite and promoting the precipitation of associated carbides. These results indicate that the microstructural effects of trace rare-earth elements are not limited to inclusion modification, but involve multiple levels of action, including grain-boundary segregation, local diffusion, and interfacial regulation.

### 2.2. Interfacial Regulation Effect of Trace Rare Earth on Microalloy Precipitates

For microalloyed steels, a particularly important issue is that trace and residual rare-earth elements not only modify inclusion types but may also directly influence the precipitation behavior of Nb, Ti, and V carbonitrides through interfacial segregation and interfacial energy restructuring. The study by Wang et al. [33] on trace Y in grain-oriented silicon steel provides important evidence in this regard. They found that trace Y promoted the precipitation of AlN. First-principles calculations further demonstrated that Y atoms tend to segregate at the Al–N interface and significantly reduce the associated interfacial energy. This suggests that rare-earth elements do not merely function by providing heterogeneous nucleation sites, but may also act as interfacially active elements that directly participate in the thermodynamic restructuring of precipitation interfaces.

The relevance of this mechanism to Nb–Ti–V microalloyed steels lies in the fact that the nucleation and growth of NbC, TiN, VC, and related carbonitrides are also strongly controlled by precipitate/matrix interfacial energy, local atomic coordination, and solute partitioning. Therefore, the Y–AlN case is used here as mechanistic evidence for rare-earth interfacial activity, while the direct connection to Nb–Ti–V precipitation is supported by reported observations such as the matrix-dependent effect of La on NbC precipitation [6,7], the low-mismatch YAlO_3_/NbC interface [36], CeAlO_3_- and Ce_2_O_2_S-induced TiN nucleation [37], and Y_2_O_3_-assisted VC precipitation [34]. Together, these studies suggest that rare-earth elements may influence microalloy precipitation through both inclusion-controlled nucleation and interface-controlled regulation.

**Figure 2:**
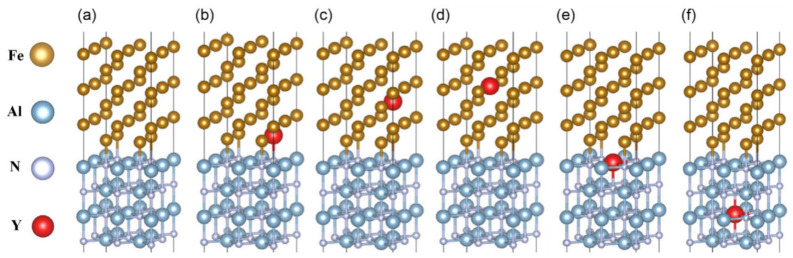
Schematic illustration of Y occupation at the Al–N interface and its interfacial regulation effect [33]. (**a**) Fe-AIN interface; (**b**) Y replace Fe at the interface; (**c**,**d**) Y replace Fe in the bulk phase; (**e**) Y replace Al at the interface; (**f**) Y replace Al in the bulk phase.

Figure 2 presents a site-occupancy model for Y at the Al–N interface and illustrates its interfacial regulation mechanism. The results indicate that rare-earth atoms preferentially occupy precipitation-related interfacial sites, thereby altering interfacial stability and precipitation sensitivity. This finding provides important insights for Nb–Ti–V microalloyed steels. If rare-earth elements segregate near austenite/precipitate interfaces, dislocation/precipitate interfaces, or grain boundaries, they may further affect the nucleation barrier, precipitation temperature, and spatial distribution of microalloy precipitates by modifying interfacial energy and local atomic coordination. Moreover, if rare-earth elements alter the local diffusion behavior of C, N, Nb, Ti, and V, their influence may extend to the coarsening and stabilization of precipitates.

Existing studies have provided further evidence for this mechanism. Gao et al. [6,7] found that the effect of La on NbC precipitation exhibits significant matrix dependence: La may inhibit NbC precipitation under austenitic conditions, whereas it may promote NbC formation in bcc Fe. Geng et al. [4] reported that Ce significantly alters the precipitation behavior of carbonitrides in the heat-affected zone of high-strength low-alloy (HSLA) steel. Chen et al. [3] further found that trace La can simultaneously modify the formation mechanisms of TiN and MnS as well as their interaction. Collectively, these studies indicate that the effect of rare-earth elements on microalloy precipitation behavior does not follow a simple and universal pattern of “promotion” or “inhibition”. Instead, it is highly dependent on interfacial conditions, the local solute environment, and the inheritance characteristics of inclusions [10,11,33].

Therefore, the influence of trace and residual rare-earth elements on microalloy precipitation behavior involves at least two coupled pathways. The first is the inclusion-inheritance pathway, in which rare-earth elements affect the heterogeneous nucleation of microalloy precipitates by modifying the type, size, and interfacial matching of inclusions. The second is the interfacial-activity pathway, in which rare-earth elements directly participate in precipitation-related interfaces in the form of solid-solution atoms, segregated species, or clusters, thereby restructuring interfacial energy and the local solute environment. The former falls within the scope of traditional inclusion metallurgy, whereas the latter is more closely related to the intrinsic microalloying effect of rare earths.

Based on this understanding, total rare-earth content alone is no longer sufficient to accurately describe the actual role of rare earths in microalloyed steels. In contrast, effective rare earth is a more appropriate evaluation parameter. It refers to the fraction of rare earths that, after deoxidation, desulfurization, inclusion formation, and solidification partitioning, can still participate in subsequent microstructural evolution in the form of solid-solution atoms, segregated species, or interfacially active components [4,5,18].

It should be emphasized that “effective rare earth” is an operational parameter rather than a single thermodynamic quantity that can be directly measured. In industrial steels, it can be experimentally estimated by combining bulk chemical analysis, inclusion extraction, automated inclusion statistics, and high-resolution interfacial characterization [10,11,15,19].

First, the total rare-earth content can be determined by inductively coupled plasma optical emission spectrometry (ICP-OES) or inductively coupled plasma mass spectrometry (ICP-MS) after complete dissolution of bulk steel samples [10,15,19]. Second, the rare-earth content fixed in stable inclusions, can be quantified by non-aqueous electrolytic extraction or selective dissolution of the steel matrix, followed by ICP-OES or ICP-MS analysis of the extracted residues. Automated scanning electron microscopy–energy-dispersive spectroscopy (SEM–EDS) inclusion analysis can further provide information on the type, size distribution, number density, and area fraction of RE-containing oxides, oxysulfides, sulfides, and composite inclusions [15,19].

For local verification, TEM/STEM–EDS and atom probe tomography can be used to identify RE segregation at grain boundaries, inclusion/matrix interfaces, and nanoscale RE-rich clusters [11,12,33].

**Figure 3:**
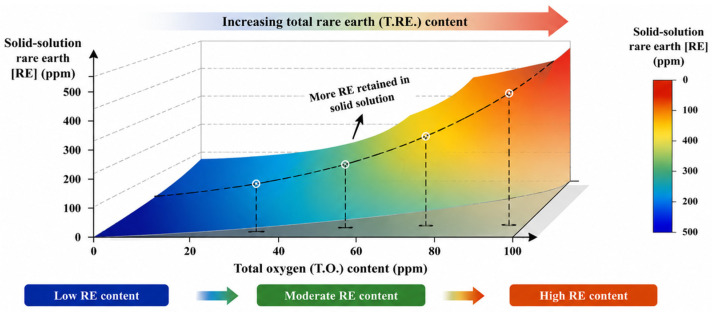
Dependence of solid-solution rare earth content on total rare earth and total oxygen contents.

Research by Chen et al. [10] indicates that the solubilized rare-earth content is controlled not only by the total rare-earth level but also by the total oxygen content. Figure 3 illustrates the relationship among solubilized rare-earth content, total rare-earth content, and total oxygen content. It can be observed that an increase in total rare-earth content does not necessarily lead to a corresponding increase in effective rare-earth content.

Based on existing research, the main occurrence states of trace and residual rare-earth elements in steels and their metallurgical significance are summarized in Table 1. Although the dominant mechanisms vary among different studies, they all point to the same essential issue: rare-earth elements are not merely compositional variables, but participate in microstructural control through the combined effects of inclusions, interfaces, and local solute environments.

## 3. How Trace and Residual Rare Earths Rewrite Inclusion Inheritance

### 3.1. Rare Earth Reconstructs Inclusion Evolution During Solidification

In studies of microalloyed steels, rare-earth elements are generally understood to affect deoxidation, desulfurization, inclusion modification, and microstructural refinement. Trace rare-earth additions can modify the composition, thermodynamic stability, and transformation sequence of inclusions during solidification. Consequently, the types of inclusions retained in the steel and available for subsequent heterogeneous nucleation are altered.

Therefore, the term “inclusion inheritance” used in this review does not simply refer to the persistence of inclusions after solidification. Rather, it refers to the ability of early-formed oxides, oxysulfides, sulfides, and composite inclusions to continue participating in microstructural evolution during subsequent cooling by acting as nuclei, coating layers, composite interfaces, or carriers of localized solute fields. In this sense, trace rare-earth elements rewrite the continuous evolution pathway of inclusions from their initial formation to their final retention.

Existing studies indicate that, after rare-earth addition, conventional deoxidation products and sulfides do not simply remain in their original forms. Instead, they may be continuously replaced, encapsulated, transformed, or selectively removed during the solidification of molten steel. For example, Liu et al. [16] found that, in low-alloy high-strength steel, inclusions evolve with increasing La content along the sequence Al_2_O_3_ and calcium aluminate → LaAlO_3_ → La_2_O_2_S → La_2_O_2_S–La_2_O_3_. Meng et al. [19] reported that, in heavy rail steel, with increasing Ce content and decreasing temperature, inclusions generally undergo restructuring along the pathway Al_2_O_3_ + MnS → CeAlO_3_ + MnS → Ce_2_O_2_S + MnS → Ce_2_O_2_S + Ce_2_S_3_ → Ce_2_O_2_S + Ce_3_S_4_ + CeS → Ce_2_O_2_S + CeS. These findings collectively demonstrate that the essential role of trace rare earths is to continuously reshape the formation sequence, stability, and retention mechanisms of inclusions before and after solidification. As a result, the inclusion system in steel may evolve from one dominated by Al_2_O_3_, MnS, and calcium aluminates to one dominated by rare-earth oxides and oxysulfides.

In conventional aluminum-deoxidized microalloyed steels, Al_2_O_3_, calcium aluminate inclusions in the CaO–Al_2_O_3_ system, MgAl_2_O_4_, and MnS typically serve as the initial substrates for subsequent inclusion evolution. Once introduced into molten steel, rare-earth elements preferentially react with dissolved oxygen, dissolved sulfur, and, in some cases, aluminum because of their strong affinity for O, S, and certain residual elements. This gradually weakens the dominant role of the original parent inclusions. Liu et al. [16] demonstrated, using automated inclusion analysis and thermodynamic calculations, that with increasing La content, inclusions in steel gradually transform from Al_2_O_3_/calcium aluminate to LaAlO_3_, La_2_O_2_S, and La_2_O_3_. Meng et al. [19] further showed that, in Ce-treated heavy rail steel, distinct phase transitions occur among CeAlO_3_, Ce_2_O_3_, Ce_2_O_2_S, and Ce sulfides as the temperature decreases from 1600 °C to 1400 °C and below.

From a thermodynamic perspective, the transformation sequence from Al_2_O_3_/MnS to CeAlO_3_, Ce_2_O_2_S, and Ce-containing sulfides should be interpreted as a shift in the stable inclusion region rather than as a simple one-step solid-state replacement reaction [15,19]. This shift is governed by temperature and by the activities of dissolved Ce, O, S, and Al in molten steel. For a general inclusion-forming reaction, the actual Gibbs free energy change can be expressed as ΔG = ΔG° + RT ln Q, where Q is determined by the activities of the reacting solutes. Therefore, even if the standard Gibbs free energy of formation of a rare-earth oxide, oxysulfide, or sulfide is negative, the actual stability of the corresponding inclusion phase depends strongly on local oxygen and sulfur activities, Ce content, Al content, and solidification segregation [15,19,34,35,36].

Thermodynamic calculations reported for Ce-treated microalloyed and rail steels support this interpretation. Zhao et al. [15] calculated the equilibrium phase regions of the Ce–Al and Ce–S systems in Q355 microalloy structural steel and showed that Ce modifies Al_2_O_3_ mainly through the formation of CeAlO_3_, whereas MnS modification is mainly associated with the formation of Ce_2_O_2_S and Ce sulfides. Representative reactions include [15]:

2[Ce] + Al_2_O_3_(s) + 3[O] = 2CeAlO_3_(s), ΔG° = 449.4T − 2.595 × 10^6^ J·mol^−1^

2[Ce] + 3MnS(s) = Ce_2_S_3_(s) + 3[Mn], ΔG° = 55.38T − 4.822 × 10^5^ J·mol^−1^

Their Ce–S equilibrium calculation further indicated that, when the S content is approximately 0.0070–0.0080 wt.%, about 173–316 ppm Ce is required to suppress MnS precipitation. This is consistent with the experimental observation that MnS was no longer detected at approximately 290 ppm Ce [15]. Similarly, Meng et al. [19] used FactSage calculations with supplemented thermodynamic data for Ce_2_O_2_S and CeAlO_3_ to show that, at 1600 °C, the stable inclusion sequence evolves from CeAlO_3_ to Ce_2_O_3_ and then to Ce_2_O_2_S with increasing Ce content. At 1400 °C and below, Al_2_O_3_ can be fully converted into CeAlO_3_ at sufficient Ce content, followed by the formation of Ce_2_O_2_S and Ce sulfides such as Ce_2_S_3_, Ce_3_S_4_, and CeS [19]. These results indicate that the apparent sequence Al_2_O_3_ → CeAlO_3_ → Ce_2_O_2_S → CeS is not universal for all steels, but depends on the thermodynamic stability fields controlled by Ce/O/S/Al activities and temperature [38].

Accordingly, the inclusion transformation discussed in this review should be regarded as a thermodynamically controlled transition between stable inclusion regions during deoxidation, desulfurization, and solidification, rather than as a purely morphological modification process.

**Figure 4:**
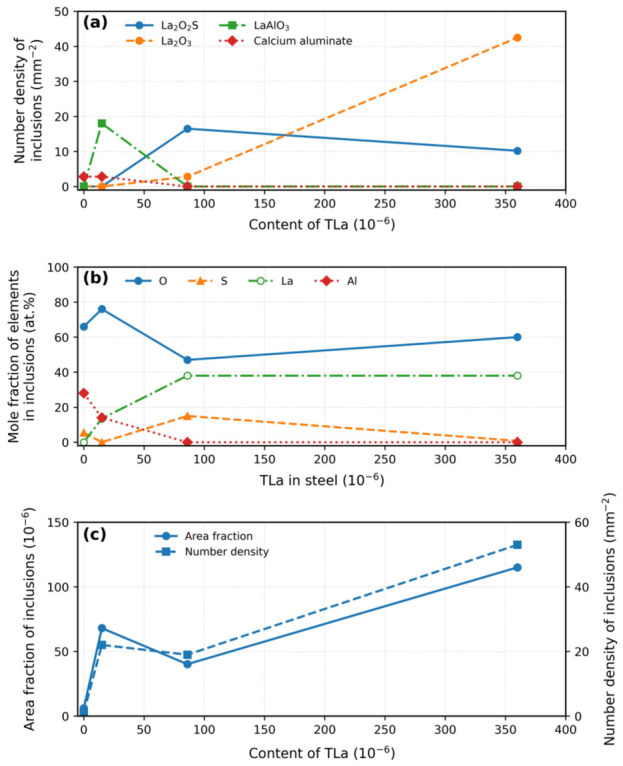
Effect of total lanthanum (TLa) content on the composition evolution, number density, and area fraction of inclusions in low-alloy high-strength steel. (**a**) kinds of inclusions; (**b**) composition; (**c**) area fraction and number density.

The La-treated low-alloy high-strength steel reported by Liu et al. [16] provides a typical example of this thermodynamically controlled inclusion reconstruction. As shown in Figure 4, increasing TLa changes the dominant inclusion type from Al_2_O_3_ and calcium aluminate to LaAlO_3_, La_2_O_2_S, and finally La_2_O_2_S–La_2_O_3_. This indicates that rare-earth addition first alters the chemical identity and stability of inclusions before affecting their nucleation capability.

**Figure 5:**
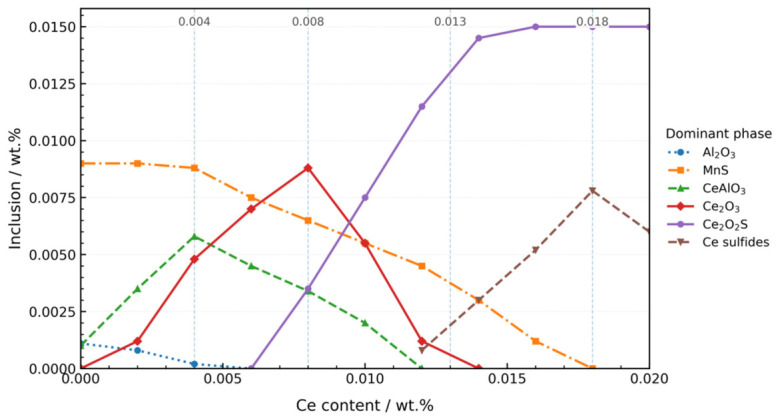
Effect of Ce content on the precipitation and transformation of inclusions in heavy rail steel at different temperatures.

Thermodynamic analysis by Meng et al. [19] indicates that distinct phase transitions occur among CeAlO_3_, Ce_2_O_3_, Ce_2_O_2_S, and Ce sulfides over different temperature ranges. As the temperature decreases, the inclusions generally continue to evolve along pathways such as Al_2_O_3_ + MnS → CeAlO_3_ + MnS → Ce_2_O_2_S + MnS → Ce_2_O_2_S + CeS. Figure 5 illustrates that trace rare-earth elements do not modify inclusions in a single step. Instead, they continuously reshape the form of inclusions as solidification progresses. In particular, within different temperature ranges, it is important to determine which inclusions can exist stably and continue to participate in subsequent microstructural evolution.

Although several studies have reported statistical information on inclusion size, number density, area fraction, and morphology after rare-earth treatment, a fully normalized statistical comparison across the literature remains difficult. The reported data were obtained from different steel grades, rare-earth addition ranges, sampling positions, analyzed areas, minimum detectable inclusion sizes, image-processing procedures, and classification criteria. In particular, inclusion number density and area fraction are highly sensitive to the selected observation area, detection threshold, and whether two-dimensional or three-dimensional statistical methods are used. Therefore, this review does not directly rank these values across different studies.

### 3.2. Effective Rare-Earth Inclusions and Their Role in Solidification Nucleation

It should be noted that not all rare-earth inclusions effectively contribute to subsequent microstructure formation. Whether an inclusion can exert a microstructural-regulating effect depends on its thermal stability, size distribution, and interfacial compatibility. Bao et al. provided typical evidence for this in their study of Fe–18Cr–0.8Si ferritic stainless steel [22]. They found that when the Ce content was only 0.012 mass pct, the improvement in the solidification microstructure was not significant. This was not because Ce itself was ineffective, but because the inclusions formed at this level were mainly large, Si-rich rare-earth inclusions. Although these inclusions were products of rare-earth reactions, they could not serve as effective heterogeneous nucleation sites for subsequent solidification microstructure formation.

**Figure 6:**
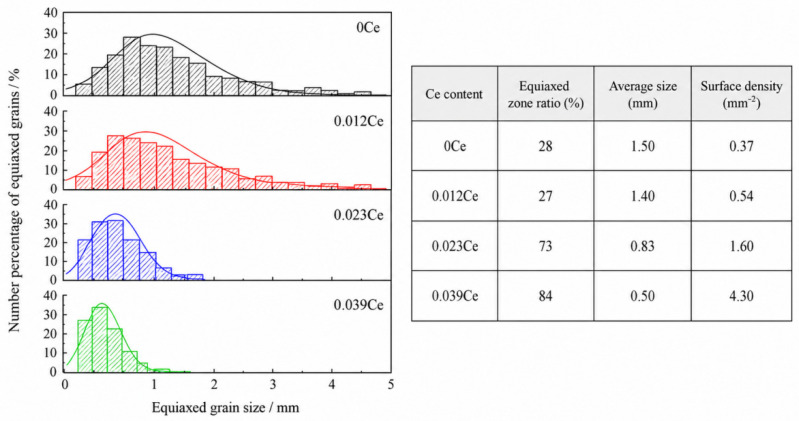
Solidification structures of Fe-18Cr-0.8Si ferritic stainless steel with different Ce contents.

In contrast, when the Ce content increased from 0.012 mass pct to 0.023–0.039 mass pct, the dominant inclusions changed from liquid or semi-liquid Ce–Si–Al–O inclusions to solid Ce–O inclusions. Most Ce–O inclusions were distributed within the size range of 1–2 μm, and the fraction of equiaxed grains increased markedly. These results indicate that the effect of rare-earth elements is not simply a matter of “the more, the better”. Rather, effective nucleation requires a suitable combination of inclusion phase constitution, size range, number density, spatial dispersion, thermal stability, and interfacial compatibility. Figure 6 demonstrates that not all rare-earth inclusions contribute equally to grain refinement. Their effectiveness as heterogeneous nucleation sites depends on factors such as phase constitution, thermal stability, particle size, and interfacial compatibility with the solidifying matrix. Only rare-earth inclusions with appropriate thermal stability, size, and interfacial conditions can serve as effective heterogeneous nucleation sites and participate in subsequent microstructure formation. This is particularly important for microalloyed steels. Because inclusions formed during solidification further influence grain-boundary distribution, local solute fields, and the interfacial conditions of subsequent precipitates, the effect of rare-earth elements on inclusion evolution does not end at the solidification stage, but continues to influence the precipitation behavior of Nb, Ti, and V carbonitrides.

Although rare-earth treatment is often discussed in terms of inclusion refinement and heterogeneous nucleation, its potential drawbacks should also be considered. Excessive rare-earth addition may promote the formation of high-melting-point rare-earth oxides, oxysulfides, and sulfides, which can agglomerate into coarse composite inclusions and reduce steel cleanliness. In Ce-treated heavy rail steel, the inclusion size first decreased and then increased with increasing Ce content, and excessive Ce caused inclusion coarsening and agglomeration, thereby negatively affecting workability, toughness, and fatigue performance [19]. In Q355 microalloy structural steel, excessive Ce also resulted in local large inclusions and non-uniform inclusion distributions, with the final inclusion size being controlled by RE content, O, S, and Al contents, as well as kinetic factors such as collision and flow conditions [15]. In addition, CeAlO_3_-rich inclusions may exhibit a strong tendency to agglomerate in molten steel and contribute to nozzle-clogging risks. Mg treatment has been reported to transform large rare-earth inclusions into more dispersed MgO-containing particles, thereby alleviating this problem. Therefore, rare-earth addition should be controlled within an optimized processing range rather than treated as a “more is better” strategy. Industrial application should also consider excessive sulfide formation, reduced cleanliness, alloying cost, addition sequence, holding time, and casting stability.

### 3.3. Residual Elements Further Complicate Inclusion Evolution

The evolution of inclusions is controlled not only by O and S, but also by various residual elements originating from raw materials and recycled steel, including As, P, Sn, Sb, and other tramp elements. Among these, As, P, and Sn have received the greatest attention in studies of rare-earth-treated steels because of their strong interactions with rare-earth elements and their significant influence on inclusion evolution. Therefore, the following discussion focuses primarily on these three representative residual elements.

The addition of trace rare-earth elements further changes the forms in which these residual elements exist during solidification. Liu et al. pointed out that, as TLa increases, the amounts of LaAlO_3_, La_2_O_2_S, and La_2_O_3_, which can serve as nucleation sites for α-Fe, gradually increase [14]. Consequently, the fraction of equiaxed grains in low-alloy high-strength steels increases from 30.1% to 50.7%, while the proportion of high-angle grain boundaries increases from 36.9% to 69.8%, promoting the formation of acicular ferrite and bainite. Bao et al. demonstrated that both increasing the number of effective nucleation particles and reducing the nucleation undercooling favor the transition from columnar grains to equiaxed grains [22]. In this regard, rare-earth elements systematically reshape the foundation of solidification microstructure formation by reconstructing the nucleation-core system available to subsequent microstructural processes. Therefore, changes in inclusions are not independent of microalloy precipitation behavior, but serve as a crucial prerequisite for the subsequent precipitation, dissolution, coarsening, and distribution evolution of Nb, Ti, and V carbides, nitrides, and carbonitrides.

**Figure 7:**
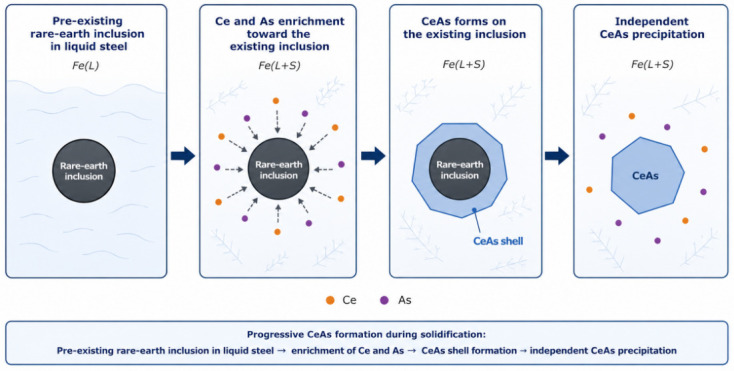
Schematic illustration of the formation mechanism of CeAs inclusions during solidification.

Research by Xin et al. on As-containing iron melts indicates that, with increasing Ce content, inclusions gradually evolve from Ce–S–O encapsulated structures to Ce–S–As and CeAs inclusions. TEM observations further confirmed that CeAs does not pre-exist in molten steel at high temperature, but forms during solidification. As shown in Figure 7, this finding is important because it shows that residual elements such as As do not simply remain as grain-boundary segregants after rare-earth addition. Instead, they may be captured by rare-earth-containing inclusions during the late stage of solidification, thereby changing both the inclusion evolution pathway and the local chemical environment near solidification fronts.

## 4. Inclusion-Controlled Precipitation Under Trace Rare Earth

This review summarizes the major pathways by which trace and residual rare-earth elements regulate precipitation behavior in microalloyed steels. The effects are not limited to a simple promotion or inhibition of precipitation, but arise from a sequence of inclusion modification, interfacial segregation, local solute partitioning, and thermomechanical-path activation.

**Figure 8:**
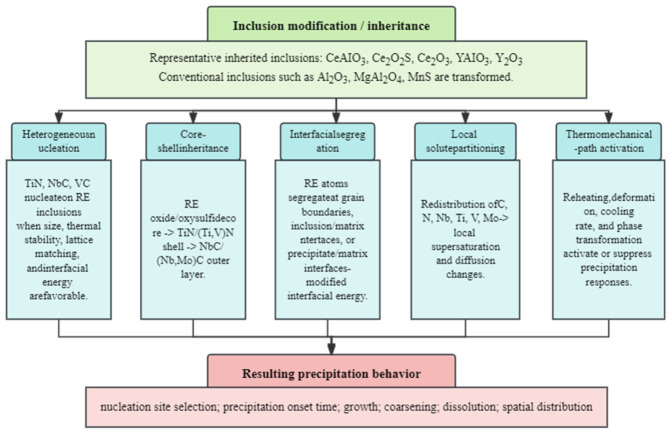
Mechanism map of rare-earth-regulated precipitation behavior in microalloyed steels.

As shown in Figure 8, rare-earth-modified inclusions may affect precipitation through several interconnected pathways, including heterogeneous nucleation, core–shell inheritance, interfacial segregation, local solute redistribution, and thermomechanical activation. The following subsections discuss these pathways in detail [39,40,41].

In microalloyed steels, the effect of trace rare-earth elements on precipitation behavior cannot be simply described as either promotion or inhibition. More accurately, rare-earth elements first modify the formation pathways, chemical composition, size distribution, and interfacial structure of inclusions. These rare-earth-modified inclusions can subsequently act as heterogeneous nucleation sites, local solute-enrichment regions, and interfacial reaction sites, thereby influencing the nucleation, growth, and spatial distribution of precipitates. Therefore, the precipitation behavior discussed in this section mainly refers to the evolution of microalloy precipitates such as NbC, TiN, VC, (Nb,Ti)(C,N), and NbC/(Nb,Mo)C, rather than to all second phases in a general sense.

In other words, the influence of rare-earth elements on precipitation behavior does not occur independently of inclusion evolution, but is realized on the basis of inclusion restructuring. When conventional inclusions such as Al_2_O_3_, MgAl_2_O_4_, and MnS are transformed into rare-earth-related inclusions such as CeAlO_3_, Ce_2_O_2_S, Ce_2_O_3_, YAlO_3_, and Y_2_O_3_, the nucleation difficulty, nucleation-site selection, and encapsulation mechanism of subsequent precipitates are all altered. Consequently, what trace rare-earth elements truly change is not simply the amount of a specific precipitate, but the formation pathway of microalloy precipitates [42,43,44,45,46,47,48].

### 4.1. Inclusion-Mediated Heterogeneous Nucleation of Microalloy Precipitates

The most direct effect of rare-earth inclusions on microalloy precipitation behavior is their role as heterogeneous nucleation sites for precipitates such as TiN, NbC, and VC. Compared with conventional Al_2_O_3_ or MgAl_2_O_4_, certain rare-earth oxides and oxysulfides exhibit lower lattice mismatch and more stable interfacial structures with microalloy precipitates, thereby significantly reducing the nucleation barrier.

Shi et al. conducted a systematic study of the YAlO_3_/NbC interface. First-principles calculations indicated that the two-dimensional lattice mismatch between YAlO_3_(001) and NbC(100) is approximately 5.4%, which falls within the effective range for heterogeneous nucleation. Further interfacial energy calculations showed that the C–Y terminated interface possesses the lowest interfacial energy, indicating high thermodynamic stability. TEM observations further confirmed that NbC can directly coat the YAlO_3_ surface to form a composite structure [30,31]. These findings indicate that rare-earth oxides not only serve as heterogeneous nucleation sites for NbC, but also facilitate NbC formation by stabilizing the interface.

**Figure 9:**
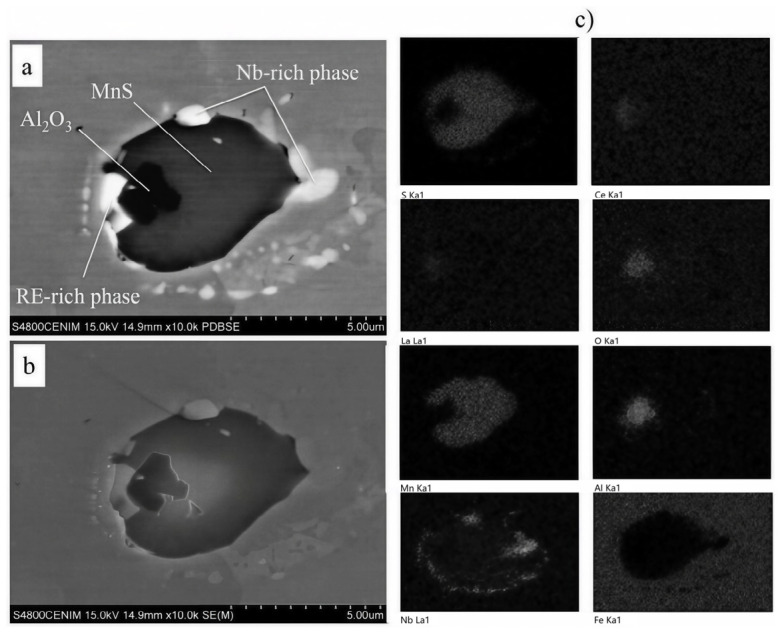
Effect of rare-earth-modified inclusions on Nb-rich precipitation behavior in Nb microalloyed steels [2]. (**a**) Back Scattered Electron; (**b**) Secondary Electron; (**c**) its elemental mappings.

Figure 9 illustrates that rare-earth addition changes the relationship between inclusions and Nb-rich precipitates mainly through modification of inclusion chemistry and interfacial conditions rather than through a simple change in Nb content. In Nb microalloyed steels, MnS-containing inclusions can promote local Nb accumulation and the formation of coarse Nb-rich carbonitrides, whereas rare-earth-modified (La,Ce)(O,S)-Al_2_O_3_ inclusions suppress this preferential Nb-rich precipitation pathway. This indicates that rare-earth treatment regulates NbC/Nb(C,N) precipitation primarily by changing the effective nucleation sites and local interfacial environment.

**Figure 10:**
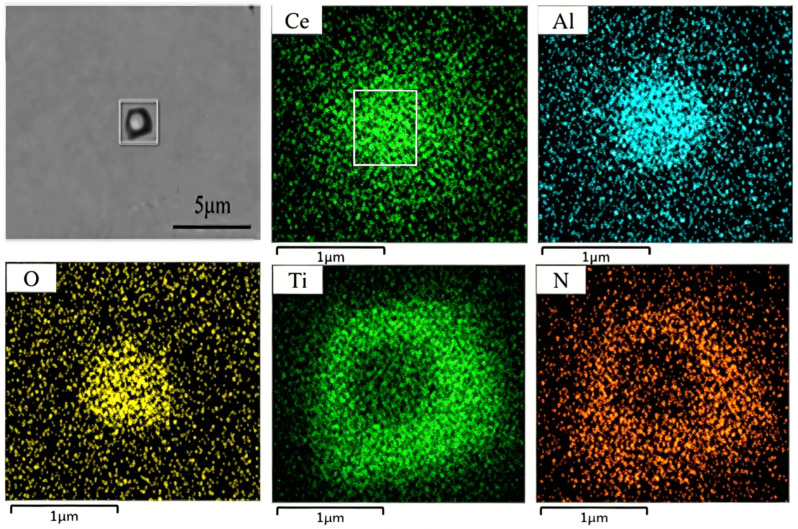
Morphological evolution and nucleation site transition of composite TiN as a function of Ce content [39].

However, the same study also showed that a further increase in Ce content does not necessarily lead to continuous optimization of TiN precipitation. Although a higher Ce content increases the number of rare-earth-bearing particles and thereby provides more potential nucleation sites, it may also promote the formation of larger Ce-rich nuclei and coarse composite TiN particles. This indicates a competition between the increase in nucleation-site density and the coarsening of nucleation cores. Therefore, the effect of rare-earth treatment on precipitation behavior should be evaluated not only by the number of available nucleation sites, but also by the size, chemistry, and interfacial stability of the rare-earth-containing cores. As schematically summarized in Figure 10, different types of rare-earth inclusions may exhibit different abilities to induce TiN formation. Compared with oxysulfides such as Ce_2_O_2_S, rare-earth oxide cores are more likely to be fully encapsulated by TiN, suggesting that inclusion chemistry and core morphology directly affect the precipitation sensitivity and final morphology of composite TiN particles.

Because lattice mismatch, or two-dimensional lattice disregistry, is an important criterion for evaluating the heterogeneous nucleation potency of inclusions, the numerical mismatch values explicitly reported for representative rare-earth-related inclusion/precipitate interfaces discussed in this review are summarized in Table 2. According to the two-dimensional lattice disregistry criterion commonly used in heterogeneous nucleation studies, a lattice disregistry below 6% is generally considered highly favorable for heterogeneous nucleation, a value between 6% and 12% indicates moderate nucleation potency, whereas a value above 12% usually suggests poor heterogeneous nucleation ability [16,19]. It should be noted that this criterion is used here as a comparative guideline, because actual nucleation potency is also affected by interfacial energy, inclusion size, chemical stability, and local solute conditions.

Only interfaces with numerical lattice mismatch values explicitly reported in the cited literature are included in Table 2. Interfaces for which only qualitative descriptions, such as “favorable matching” or “low mismatch”, were provided are discussed in the text but are not included in the comparative table.

### 4.2. Hierarchical Core–Shell Precipitation and Inheritance Effects

Compared with single-step heterogeneous nucleation, the influence of rare-earth elements on hierarchical precipitation behavior warrants greater attention. In many microalloyed steels, precipitation does not occur in isolation but exhibits distinct core–shell succession characteristics, in which inclusions or precipitates formed at an earlier stage continue to serve as nuclei for subsequent precipitation [41]. Lu et al. observed a typical three-layer structure in Y-containing 11Cr ferritic/martensitic (F/M) steel: YAlO_3_–TiN/(Ti,V)N–NbC/(Nb,Mo)C. Their results showed that YAlO_3_ forms first, followed by the precipitation of TiN on its surface, while NbC further grows epitaxially on the outer TiN layer [42]. Because of the low lattice mismatch between YAlO_3_ and TiN, as well as between TiN and NbC, a stable multilayer precipitation structure is formed.

**Figure 11:**
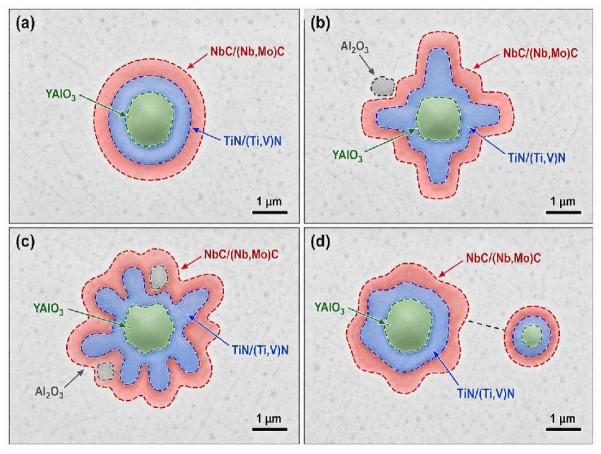
The rare-earth oxide core can sequentially induce the precipitation of TiN/(Ti,V)N and NbC/(Nb,Mo)C layers. (**a**) concentric core–shell structure; (**b**) Cross-shaped multilayer precipitate formed on a YAlO_3_core; (**c**) Irregular star-like multilayer precipitate with a YAlO_3_core; (**d**) Irregular multilayer precipitate centered on a YAlO_3_core.

Figure 11 illustrates that rare-earth-induced changes in inclusion composition and interfacial characteristics not only alter the nucleation conditions for individual precipitation events, but may also influence the subsequent precipitation sequence. In other words, once the composition and interfacial characteristics of the initial inclusion core are modified by rare-earth treatment, the evolution pathway of the subsequent multilayer precipitation structure may also change. A similar phenomenon has been reported in H13 steel. Huang et al. [43] found that, in the absence of Ce, MgAl_2_O_4_ can act as a nucleus for multilayer (Ti,V)(C,N) precipitation. After Ce addition, CeAlO_3_ can still induce multilayer carbonitride precipitation. However, with further increases in Ce content, the dominant inclusions gradually transform into Ce–O and Ce–O–S phases, and the multilayer precipitation structure becomes significantly less pronounced.

These results indicate that rare-earth elements do not simply increase the number of nucleation sites, but instead rewrite precipitation pathways by changing the type of nucleation site. When the nucleation sites remain low-mismatch CeAlO_3_, multilayer precipitation can be maintained. However, when the nucleation sites transform into high-mismatch Ce–O/Ce–O–S inclusions, the original epitaxial precipitation pathways are weakened or even interrupted.

### 4.3. Promotion, Suppression and Pathway Switching

**Figure 12:**
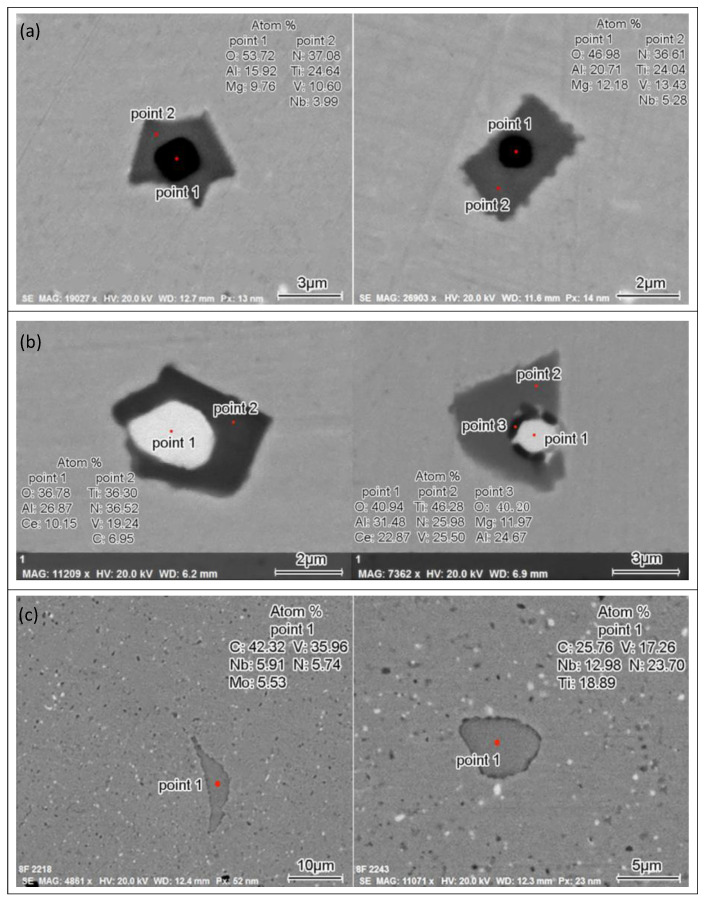
Effect of Ce on the morphology of multilayer carbonitrides in H13 steels [43]. (**a**) Core composed of MgAl_2_O_4_; (**b**) Core transformed intoCeAlO_3_; (**c**) Single-phase carbonitride appears, corresponding to the Ce-O/Ce-O-S core.

One of the most common misconceptions regarding the effect of rare-earth elements on precipitation behavior is the assumption that, because rare-earth inclusions can serve as nucleation sites, they necessarily promote precipitation. In fact, existing studies have shown that trace rare-earth elements exert a strong competitive effect on precipitation behavior, and the final response depends on the interaction among inclusion type, size distribution, interfacial structure, and solidification microstructure. Huang et al. [43] demonstrated in H13 steel that both MgAl_2_O_4_ and CeAlO_3_ can induce the precipitation of multilayer (Ti,V)(C,N) phases. However, when the inclusions further transform into Ce–O and Ce–O–S phases, the multilayer carbonitride structure is significantly reduced (Figure 12). This suggests that rare-earth elements do not always promote precipitation, but may instead switch the precipitation pathway by changing the core type.

Ren et al. [44] further found that Ce_2_O_3_ and Ce_2_O_2_S promote the nucleation of VC and Mo_2_C, resulting in an increase in the area fraction of primary carbides under low-Ce conditions. However, as the Ce content continues to increase, significant refinement of the dendritic structure compresses the space available for carbide growth, ultimately causing the carbide area fraction to decrease. These results indicate that, in the low-Ce range, the process is primarily controlled by the heterogeneous-nucleation promotion effect, whereas in the high-Ce range, it gradually becomes dominated by microstructural-refinement-induced suppression. The competition between these two effects leads to a distinctly non-monotonic change in precipitation behavior.

Research by Jiang et al. [45] on high-nitrogen interstitial-free (IF) steels also suggests that an increase in the number of nuclei does not necessarily imply optimized precipitation. Although Ce treatment alone increased the number of TiN nuclei, severe aggregation of Ce-rich nuclei caused multiple TiN particles to precipitate on the same coarse nucleus, ultimately forming coarse TiN clusters. In contrast, combined Mg–Ce treatment suppressed the coalescence of Ce-rich nuclei, allowing TiN to achieve true refinement and dispersion.

As shown in Table 3, when evaluating whether rare-earth elements promote precipitation, it is necessary to simultaneously consider the number of nuclei, nucleus size, spatial distribution of nuclei, degree of interfacial matching, and solidification microstructure scale. Only when these factors are optimized together does the promoting effect of rare-earth elements have practical microstructural significance.

### 4.4. Beyond Inclusion Nucleation: Interfacial Segregation and Local Partitioning

Although heterogeneous nucleation on inclusions can explain the precipitation of large amounts of TiN, NbC, and VC, it is insufficient to account for certain more complex observations. For example, in some steel grades, significant changes in precipitation behavior occur even when typical rare-earth inclusion nuclei are not observed. This suggests that the role of trace rare-earth elements is not limited to geometric nucleation, but also involves interfacial segregation, local compositional rearrangement, and interfacial energy restructuring. Chen et al. [46] found that, in Y-modified H13 steel, Y_2_O_3_ not only induces the nucleation of VC and Cr_23_C_6_, but also leads to the formation of distinct Y-enriched layers, stacking faults, and interfacial interdiffusion near the Y_2_O_3_/Cr_23_C_6_ interface. This indicates that rare-earth inclusions can provide active interfaces capable of dynamically regulating local diffusion and interfacial stress.

Similarly, Zhuang et al. [47] reported that, in 15–15Ti–Y steel, Y segregation at grain boundaries weakens the tendency of M_23_C_6_ to precipitate at grain boundaries while increasing the proportion of intragranular precipitation. This result indicates that even when rare-earth elements do not act directly as precipitation nuclei, they can still reshape precipitation sensitivity by competing for grain-boundary sites and modifying local solute distribution and interfacial energy. The study by Yang et al. [48] on 316LN steel further demonstrates that the inclusion-core effect and the solid-solution rare-earth effect can coexist synergistically. On the one hand, Ce-rich TiN nanoparticles can serve as nucleation sites for the Laves phase; on the other hand, solid-solved Ce can attract surrounding Mo atoms, increase the local Mo concentration, and thereby promote more uniform precipitation of the Laves phase.

The effects of different rare-earth mechanisms can be categorized into three scenarios: inclusion-controlled mechanisms, solute rare-earth mechanisms, and segregation-controlled mechanisms. Inclusion-controlled mechanisms refer to cases in which rare-earth-containing oxides, oxysulfides, sulfides, or composite inclusions serve as physical nucleation substrates for TiN, NbC, VC, or related carbonitrides. The supporting evidence typically includes direct observation of precipitates attached to rare-earth inclusions, core–shell structures, lattice mismatch calculations, or interfacial energy analysis.

Solute rare-earth mechanisms refer to cases in which rare-earth elements are retained in the matrix as dissolved atoms or nanoclusters and influence precipitation by altering solubility, diffusion behavior, or local solute distribution, without necessarily acting as visible inclusion nuclei. Typical examples include the matrix-dependent effects of La on NbC precipitation in austenite and ferrite/body-centered cubic Fe, as well as the ability of solid-solved Ce to attract Mo atoms and modify Laves phase precipitation.

Segregation-controlled mechanisms refer to cases in which rare-earth elements are enriched at grain boundaries, precipitate/matrix interfaces, or inclusion/matrix interfaces, thereby altering interfacial energy, grain-boundary site competition, or local precipitation sensitivity. Representative examples include Y segregation at the Al–N interface, Y-enriched interfacial layers near the Y_2_O_3_/Cr_23_C_6_ interface, and Y segregation at grain boundaries affecting M_23_C_6_ precipitation [42,43,44,45,46,47,48,49,50].

Based on this classification, the major mechanisms by which trace rare-earth elements regulate microalloy precipitation behavior through inclusions, solute effects, and interfacial segregation are summarized in Table 4. It should be emphasized that the role of rare-earth elements does not follow a single fixed mechanism. Rather, depending on the occurrence state of rare earths, inclusion type, interfacial structure, and local solute environment, heterogeneous nucleation promotion, precipitation pathway switching, interfacial energy restructuring, and local diffusion regulation may compete or operate synergistically.

## 5. Why the Reported Precipitation Responses Are Contradictory

The precipitation behavior discussed in this review specifically refers to the nucleation, growth, coarsening, dissolution, and spatial distribution of carbides, nitrides, and carbonitrides formed by microalloying elements such as Nb, Ti, and V under different thermomechanical pathways. It does not include the formation of inclusions in the matrix. Previous studies have shown that substantial disagreement has long existed regarding the precipitation behavior of microalloyed steels. Some studies suggest that deformation can significantly promote precipitation and advance the onset time, whereas others indicate that, once recrystallization occurs preferentially, precipitation kinetics slow down markedly. Some works predict, based on thermodynamic analysis, that stable precipitation should occur within specific temperature ranges, yet no significant precipitates are observed experimentally even after prolonged holding. Furthermore, steels with the same V content may exhibit either regular interphase precipitation or dislocation/grain-boundary-dominated random precipitation, depending on the heat-treatment path [51,52,53,54,55,56,57].

These discrepancies indicate that precipitation promotion and inhibition are not absolute concepts. Instead, they depend on the specific parameters being compared, such as precipitation onset time, peak precipitation temperature range, precipitate size, number density, volume fraction, degree of solute depletion, or contribution to precipitation hardening [53,54,55,56]. Unless the observation scale and evaluation criteria are clearly specified, conflicting results reported in different studies do not necessarily constitute true contradictions.

More importantly, microalloy precipitation does not occur independently. Rather, it interacts with recrystallization, dislocation evolution, interface migration, phase transformation, and local solute redistribution during thermomechanical processing. For example, strain-induced precipitation may retard recrystallization by pinning grain boundaries, whereas recrystallization can alter the density of nucleation sites and the diffusion pathways available for precipitation. Therefore, many seemingly contradictory results essentially correspond to different controlling stages in the precipitation process. For trace rare-earth systems, this complexity is further amplified because rare earths not only alter the type of precipitates but may also simultaneously modify interfacial states, local diffusion pathways, and precipitation sensitivity. Consequently, the so-called “rare-earth-promoted precipitation” and “rare-earth-inhibited precipitation” reported in the literature often correspond to different dominant control mechanisms rather than representing a true direct contradiction.

**Figure 13:**
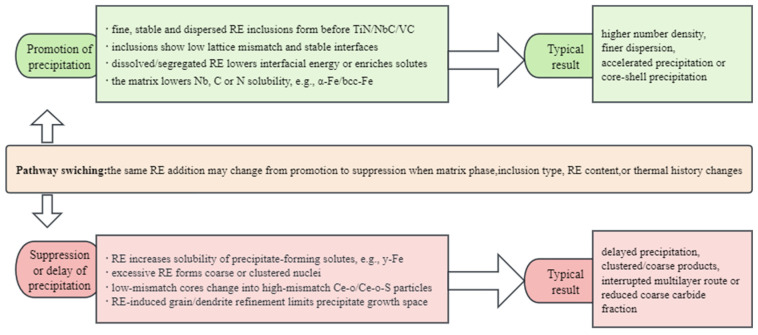
The metallurgical conditions under which rare earths promote, suppress, or redirect precipitation in microalloyed steels.

To provide a clearer explanation of this issue, the condition-dependent framework shown in Figure 13 is proposed. Rare-earth elements tend to promote precipitation when they form fine, stable, and well-dispersed inclusions that act as low-mismatch heterogeneous nucleation sites for TiN, NbC, VC, and related carbonitrides, or when dissolved or segregated rare-earth atoms reduce interfacial energy and locally enrich precipitate-forming solutes. In contrast, rare-earth elements may suppress or delay precipitation when they increase the solubility of precipitate-forming elements in the matrix, form coarse or clustered RE-rich nuclei, transform low-mismatch nuclei into high-mismatch Ce–O or Ce–O–S particles, or refine the solidification structure and thereby restrict precipitate growth.

Representative observations reported in the literature are summarized in Table 5. By distinguishing the dominant kinetic stage affected, Table 5 shows that rare-earth additions may influence precipitation at different stages, including nucleation, early growth, coarsening, dissolution or solubility control, and precipitation pathway selection. It can also be seen that the same rare-earth element may produce markedly different precipitation responses depending on steel composition, rare-earth concentration, inclusion characteristics, matrix state, and thermomechanical processing route. Therefore, the apparent contradictions reported in different studies should not be interpreted as mutually exclusive conclusions. Instead, they reflect different kinetic stages and controlling factors within a path-dependent precipitation process.

### 5.1. Contradiction Is Intrinsic to a Path-Dependent Process Rather than Experimental Scatter

Conflicting reports on precipitation behavior are common primarily because precipitation is, by nature, a strongly path-dependent non-equilibrium process. The classic study by Hansen et al. demonstrated that Nb(C,N) precipitation and austenite recrystallization are not independent parallel processes, but coupled processes that continuously compete with and influence each other. If unrecrystallized austenite is retained, grain boundaries, deformation zones, dislocation cells, and substructures can provide a high density of heterogeneous nucleation sites, thereby significantly accelerating precipitation. Conversely, if recrystallization is completed first, these high-defect-density nucleation sites disappear, and the precipitation kinetics slow down markedly.

Dutta et al. [52] further pointed out that strain-induced precipitation is not merely an acceleration of nucleation. Dislocation-channel diffusion can also cause precipitates to enter a regime in which nucleation, growth, and coarsening occur simultaneously at an early stage. Consequently, even when different studies report enhanced precipitation, the underlying microscopic mechanisms may be fundamentally different. In one case, enhanced precipitation may be reflected by a significant increase in number density and a small precipitate size, whereas in another case, simultaneous growth and coarsening may occur, causing the number density to begin decreasing. Therefore, the apparent contradiction in the literature between enhanced and reduced precipitation often does not originate from thermodynamics itself, but from the fact that the investigated systems are located at different stages of the precipitation evolution trajectory.

**Figure 14:**
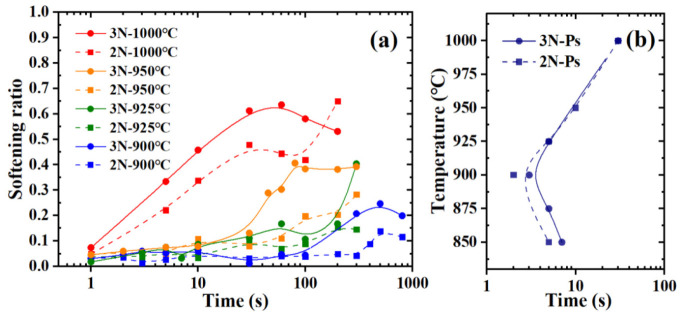
Schematic comparison of precipitation kinetics in strain-free and strained austenite. (**a**) strain Free: the precipitates are (Nb,Ti)C at all temperatures; (**b**) strained: The precipitates are (Nb,Ti)C at 950 °C and 1000 °C,and (Nb,Ti,V)C at 850 °C and 900 °C.

As shown in Figure 14, precipitation kinetics in Nb–Ti–V microalloyed steels are strongly affected by the deformation state of austenite. Compared with strain-free austenite, deformed austenite generally exhibits a shorter precipitation incubation period and a faster precipitation response, because deformation introduces high-density dislocations and substructures that provide additional nucleation sites and fast diffusion paths. Moreover, the dominant precipitate chemistry may vary with the temperature window. At relatively high temperatures, Nb- and Ti-rich carbonitrides are more likely to dominate, whereas at lower temperatures, V-containing carbonitrides may become more evident as the solubility and diffusion conditions change. Therefore, conclusions regarding precipitation promotion or delay cannot be directly compared unless the deformation state, defect density, holding temperature, and precipitate type are considered together.

### 5.2. Influence of Pre-Precipitation Austenite State on Precipitation Behavior

Another key variable affecting precipitation behavior is the prior austenite condition, particularly the recrystallization state, dislocation density, and distribution of crystal defects before precipitation. This issue is especially important in microalloyed steels because deformation modifies not only the density of effective nucleation sites, but also the subsequent competition between precipitation and recrystallization. Tsao et al. [53] demonstrated in Nb microalloyed steels that strain-induced precipitation of NbC is highly sensitive to deformation temperature and holding time, and that deformation significantly accelerates precipitation by introducing dislocations and other crystal defects that provide favorable nucleation sites and fast diffusion paths. Zhang et al. [54] further showed in Ti microalloyed steel that the apparent precipitation response is strongly coupled with recrystallization behavior: when unrecrystallized austenite is retained, precipitation occurs more readily, whereas when recrystallization proceeds preferentially, the onset of precipitation is delayed. These findings indicate that the prior austenite state fundamentally governs the subsequent precipitation response, and that precipitation behavior cannot be interpreted solely in terms of alloy composition or equilibrium thermodynamics.

In addition to deformation and recrystallization, the reheating temperature and holding time before deformation are also critical variables because they determine the dissolution state of pre-existing TiN, NbC, and complex (Ti,Nb)(C,N) particles. When the reheating temperature or holding time is insufficient, undissolved TiN or (Ti,Nb)(C,N) particles may remain in the austenite. These particles reduce the effective solute contents of Ti, Nb, C, and N available for subsequent precipitation, while also acting as inherited heterogeneous nucleation substrates during cooling or deformation. In contrast, higher reheating temperatures or longer holding times may increase the dissolved Nb and Ti contents, thereby enhancing supersaturation during subsequent cooling, but may also reduce the number of pre-existing nucleation cores. Therefore, differences in reheating history can lead to markedly different precipitation responses even in steels with similar nominal compositions.

Similarly, when comparing Nb–V and Ti–V microalloying systems, Pandit et al. [57] noted that the strain-induced precipitation kinetics of the two systems exhibit significant differences. The root cause does not lie in a change in the role of V, but rather in the low solubility of Ti in austenite and the presence of undissolved TiN, which significantly reduce the effective solute Ti content and further alter the subsequent precipitation pathways of composite carbonitrides. Therefore, if two studies employ different reheating regimes, holding temperatures, and holding times, they actually correspond to different initial solute states and inherited grain states. Consequently, their precipitation responses should not be directly compared side by side.

**Figure 15:**
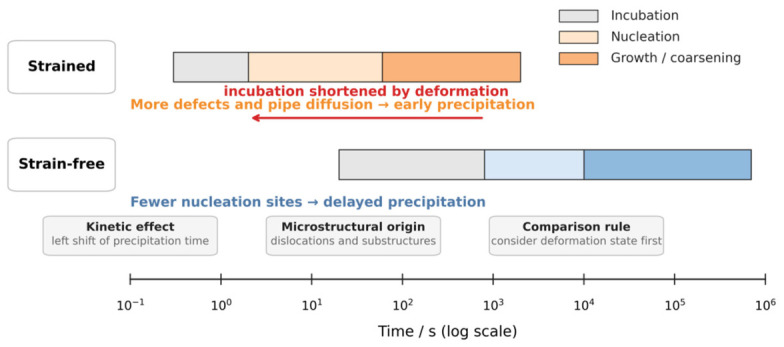
Conceptual comparison of precipitation response in strain-free and strained austenite.

**Figure 16:**
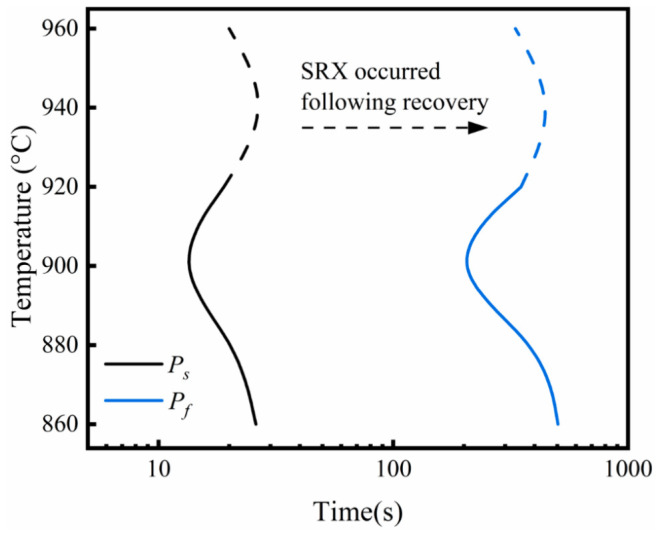
Open-access example of strain-induced precipitation kinetics in microalloyed steel, represented by a precipitation–time–temperature curve obtained after controlled compression [54].

Figure 15 and Figure 16 further illustrate that deformation-promoted precipitation is not merely an empirical observation, but a consequence of changes in the metallurgical state of austenite before precipitation. Deformation increases the density of dislocations and substructures, shortens the precipitation incubation period, and modifies the subsequent precipitation–recrystallization interaction. Consequently, if strain-free and deformed austenite are not clearly distinguished, or if the defect state before and after recrystallization is not specified, the precipitation-start time, nose temperature of the PTT curve, and the apparent precipitation kinetics may be interpreted in contradictory ways. This is one of the major reasons why different studies may report apparently inconsistent conclusions regarding precipitation promotion or delay.

### 5.3. Thermodynamic Permissibility Does Not Guarantee Kinetic Accessibility

The fact that precipitation is thermodynamically permitted does not necessarily mean that it will occur within the experimental time scale. This is particularly evident in V-microalloyed steel systems. A combined study by Ioannidou et al., using dilatometry, small-angle neutron scattering (SANS), and atom probe tomography (APT), demonstrated that although thermodynamic equilibrium calculations predicted VC precipitation at 900, 750, and 650 °C, no clear precipitation was observed experimentally even after holding the samples at 900 and 750 °C for up to 10 h. Detectable VC precipitation occurred mainly at 650 °C and became significant only after the initiation of the austenite-to-ferrite transformation [58].

This result indicates that determining whether precipitation occurs within a given temperature range cannot rely solely on equilibrium phase diagrams or theoretical equilibrium volume fractions. Instead, it is necessary to simultaneously consider diffusion rates, interfacial migration, phase-transformation-induced local solute redistribution, and kinetic accessibility within a finite time scale. Therefore, while some studies emphasize the suppression of precipitation in the high-temperature region, others highlight enhanced precipitation in the low-temperature region. These findings are not contradictory, because they essentially correspond to the upper limit of thermodynamic stability and the range of kinetic accessibility within the experimental time scale, respectively.

### 5.4. Phase Transformation Rewrites the Precipitation Range

Phase transformation is not a secondary process following precipitation; rather, it actively redefines the precipitation range. In V-microalloyed steels, the γ → α phase transformation not only alters the equilibrium solubility of solutes in the two phases, but also simultaneously changes the effective nucleation sites and the local diffusion field. Research by Ioannidou et al. indicated that, at 650 °C, VC precipitation occurs mainly after the phase transformation has started [58]. The evolution of precipitate average radius, number density, and volume fraction differs before and after phase transformation: the early stage is dominated by nucleation and growth, whereas after completion of the phase transformation, the process gradually shifts to a coarsening-controlled stage, during which the number density may actually decrease.

The phase-transformation-controlled precipitation behavior discussed here is particularly relevant to VC or V-rich carbonitrides, because V has relatively high solubility in austenite and VC precipitation often becomes kinetically accessible during or after the γ → α transformation. NbC or Nb(C,N) can also be affected by the γ → α transformation through changes in solubility, dislocation density, and local solute partitioning, but Nb-containing precipitates may additionally form in deformed or unrecrystallized austenite through strain-induced precipitation. Therefore, their precipitation range is controlled by both prior austenite state and transformation path. TiN is different again: because of its high thermodynamic stability and very low solubility, TiN commonly forms at high temperature during solidification or reheating and may be inherited before the ferrite transformation occurs. Thus, the phase-transformation argument should not be generalized equally to VC, NbC, and TiN. Instead, it should be understood as precipitate-specific: VC is often strongly transformation-coupled, NbC is jointly controlled by deformation/recrystallization and transformation, whereas TiN is mainly governed by high-temperature formation, dissolution resistance, and inheritance effects.

Subsequent in situ neutron diffraction and simultaneous SANS studies further indicated that increasing V and C contents can enhance the final number density of precipitates, while simultaneously delaying the γ → α phase transformation itself. Therefore, alloying elements do not promote precipitation simply by increasing supersaturation; they also alter the initiation time and progression rate of phase transformation, thereby further affecting the timing, location, and mechanism of precipitation. Consequently, while some studies emphasize that high V/C ratios increase the number of precipitates, others report that high V/C ratios delay the onset of precipitation. In reality, these findings correspond to two different dimensions: the final precipitation yield and the timing of precipitation initiation.

**Figure 17:**
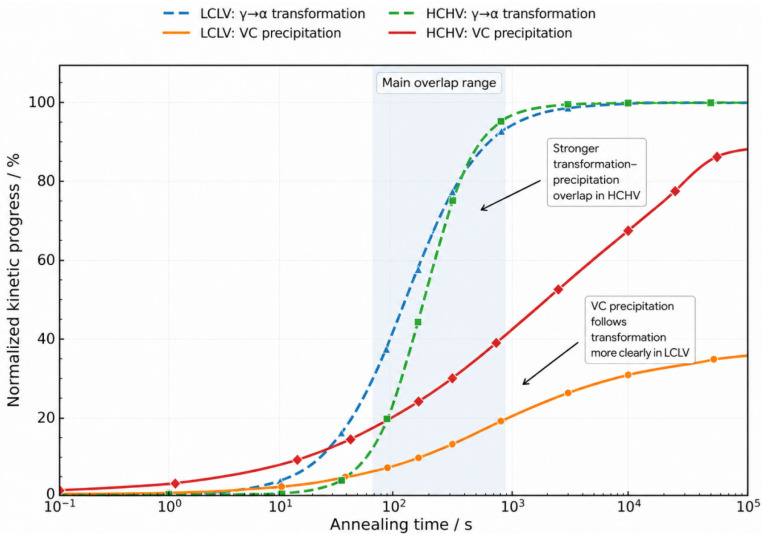
Vanadium carbide precipitation and austenite-to-ferrite phase transformation kinetics during isothermal annealing at 650 °C in the low-carbon–low-vanadium alloy (LCLV) and high-carbon–high-vanadium alloy (HCHV) steels.

As shown in Figure 17, there is no simple linear relationship between the phase transformation fraction and the precipitate volume fraction; the actual onset of precipitation is often controlled by the phase transformation process. Consequently, discussing the promotion or inhibition of precipitation in isolation from the phase transformation pathway can easily lead to apparently contradictory conclusions. As shown in Table 6, such apparent contradictions often arise from differences in precipitation range, microstructural state, and evaluation criteria. This issue is particularly important for trace rare-earth systems, because rare-earth elements influence precisely these hidden state variables.

### 5.5. Interphase Precipitation and Random Precipitation Are Not Equivalent Strengthening States

Even within the same alloy system, the precipitation mechanisms discussed in different studies may differ. A SANS study by Khan et al. [63] on nanoscale V-alloyed steels demonstrated that different heat-treatment paths can lead to the formation of interphase precipitation and random precipitation, respectively. These two types differ not only in their spatial arrangement, but also exhibit systematic differences in number density, size distribution, preferred nucleation sites, and strengthening mechanisms. Interphase precipitation typically exhibits a higher number density and a more regular lamellar arrangement, whereas random precipitation tends to form near grain boundaries, subgrain boundaries, and dislocations, and displays a broader size distribution.

From a strengthening perspective, regular interphase precipitation usually forms nanoscale particles arranged in sheet-like rows with relatively small and periodic interparticle spacing. Such a distribution is favorable for precipitation strengthening because dislocation bypassing is strongly affected by particle spacing and number density. However, its strengthening contribution depends on the continuity, spacing, and thermal stability of the interphase sheets. In contrast, random precipitation on dislocations, subgrain boundaries, or grain boundaries generally produces a less periodic particle distribution and a broader size range. Fine random precipitates on dislocations can still contribute effectively to yield strength, whereas coarse grain-boundary precipitates or over-coarsened particles usually provide less uniform strengthening and may reduce grain-boundary toughness. Therefore, interphase precipitation and random precipitation cannot be evaluated using only precipitate volume fraction; particle spacing, coarsening resistance, spatial distribution, and the corresponding contribution to yield strength must also be considered.

**Figure 18:**
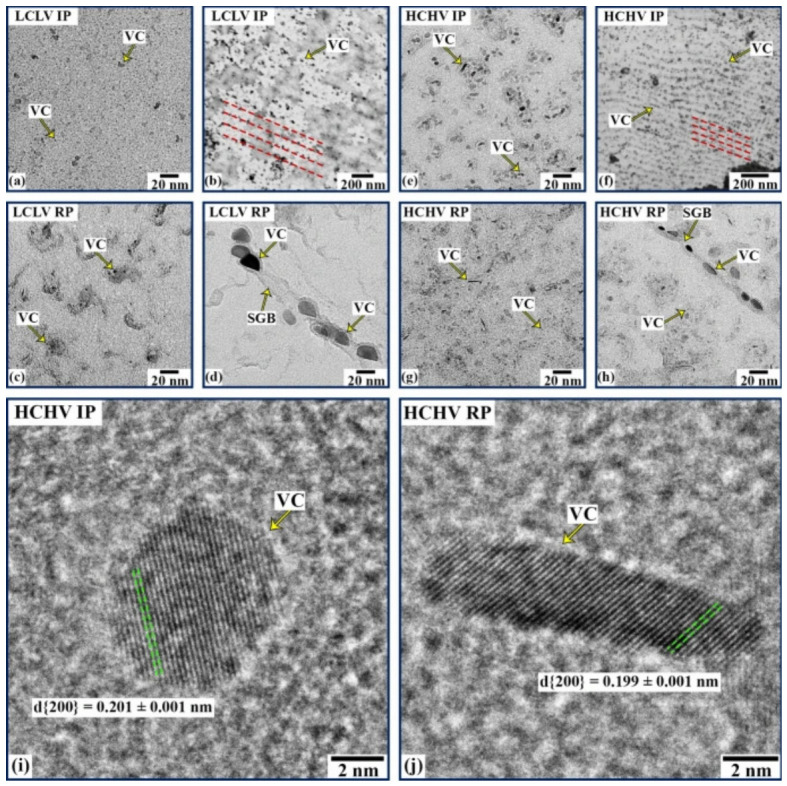
Morphological contrast between interphase precipitation and random precipitation of nanoscale VC precipitates in vanadium micro-alloyed steels [63]. Bright-field TEM images of the CERs for the (**a**,**b**) LCLV IP (interphase precipitation), (**c**,**d**) LCLV RP (random precipitation), (**e**,**f**) HCHV IP and (**g**,**h**) HCHV RP samples. (**i**,**j**) HRTEM micrographs of the CERs illustrating lattice images of VC nano-precipitates for the HCHV IP and HCHV RP samples, respectively. SGB: sub-grain boundary of bainite/martensite.

Therefore, the seemingly opposite precipitation responses reported in the literature are often not due to opposite thermodynamic tendencies, but because the systems under investigation actually belong to different precipitation mechanisms. Figure 18 demonstrates that once the thermal path changes, the system is no longer in the same precipitation state. Consequently, any comparison of precipitation promotion or inhibition must first clarify whether it corresponds to interphase precipitation, random precipitation, or a composite state in which both coexist.

### 5.6. Different Characterization Methods Observe Different Segments of the Same Precipitation Trajectory

Stress relaxation and electrical resistivity methods are generally more sensitive to the onset of precipitation and the corresponding softening or hardening responses. Therefore, they are well suited for capturing the precipitation-start time, nose-tip temperature, and kinetic acceleration associated with strain-induced precipitation. TEM is more suitable for directly observing local nucleation sites, precipitate morphology, and orientation relationships; however, its statistical sampling volume is limited, which may lead to overemphasis on localized features. APT is highly sensitive to atomic-scale compositional gradients, but it is less suitable for directly determining precipitate volume fraction on a macroscopic statistical scale. SANS can reliably provide average radius, number density, volume fraction, and size distribution, but it generally requires complementary TEM or APT observations to constrain precipitate shape and composition.

Therefore, as summarized in Table 7, it is entirely possible for the same system to show apparently inconsistent results when different characterization methods are used. For example, TEM may reveal a large number of fine precipitates, while SANS indicates that the overall volume fraction remains low. APT may show that precipitate cores approach stoichiometric composition, while electrical resistivity still suggests that solute depletion is incomplete. Similarly, stress relaxation may detect the onset of precipitation before conventional TEM can resolve the precipitates. These discrepancies should not be interpreted as contradictions. Rather, they reflect the fact that different characterization techniques capture different stages and length scales along the same precipitation evolution trajectory.

## 6. A Conceptual Framework for Inclusion–Precipitation Coupling in Microalloyed Steels

Existing research on the role of rare-earth elements in microalloyed steels has generally followed two relatively independent paths. One focuses on inclusion engineering, emphasizing the effects of inclusion composition, size, and number density on solidification microstructure and intragranular nucleation. The other focuses on the precipitation kinetics of Nb, Ti, and V carbonitrides, centering on recrystallization–precipitation competition, precipitation–time–temperature (PTT) curves, recrystallization–precipitation–time–temperature (RPTT) curves, and precipitation behavior during continuous cooling [63,64,65,66,67,68]. However, in microalloyed steels, inclusion evolution and microalloy precipitation are not mutually exclusive issues. What enters the subsequent hot-working and cooling stages is not an isolated inclusion or a homogeneous solid solution, but a heterogeneous system inherited from solidification, characterized by specific interfacial structures and local chemical environments [69]. Therefore, these apparently separate phenomena should not be unified through a single narrative of either inclusion modification or precipitation kinetics alone, but through an inclusion–precipitation coupling framework centered on interfacial states.

From the perspective of solidification metallurgy, inclusions do not remain unchanged once they form in molten steel. Instead, they continue to evolve under the combined effects of microsegregation, interfacial reactions, and diffusion kinetics. Existing studies indicate that the inclusion spectrum formed and inherited during solidification further influences the precipitation of sulfides, nitrides, and carbides in the solid state, thereby linking the traditionally separate domains of molten-steel purification and final microstructural properties [70,71,72,73,74]. In other words, the starting point for subsequent precipitation behavior is not an ideal homogeneous solid solution, but a non-homogeneous matrix characterized by inherited inclusions and local segregation. For trace rare-earth systems, even extremely low concentrations of rare earths can cause systematic deviations in subsequent precipitation behavior by altering the phase composition, core–shell structure, size distribution, or interfacial properties of inherited inclusions.

**Figure 19:**
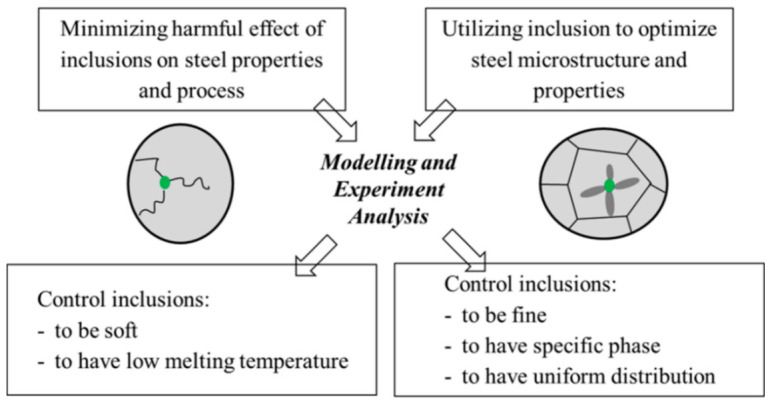
Concept of inclusion engineering and its implication for subsequent microstructure optimization during steel solidification and cooling [68].

**Figure 20:**
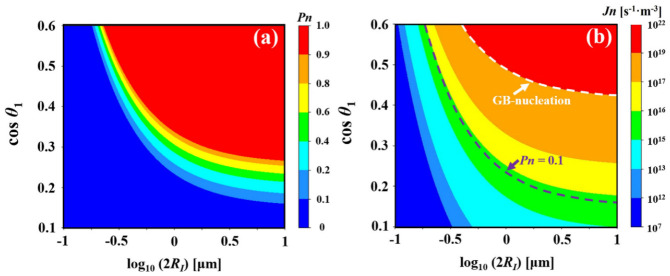
Interaction map showing the coupled effect of inclusion size and interfacial energies on heterogeneous nucleation potency [70]. (**a**) acicular ferrite-nucleation probability, Pn; (**b**) acicular ferrite-nucleation rate, Jn.

As shown in Figure 19, the core of inclusion engineering lies not merely in reducing the number of harmful inclusions, but more importantly in modifying their subsequent microstructural effects by controlling their composition, size, and interfacial structure. For microalloyed steels, this means that the state of inclusions formed during solidification directly influences the subsequent precipitation conditions of Nb, Ti, and V carbonitrides. Figure 20 further illustrates that the effectiveness of inclusions is not determined solely by size or chemical composition, but is jointly controlled by interfacial energy, lattice matching, and size effects. Extending this concept to microalloyed steels helps explain why even rare-earth-modified inclusions can produce significantly different effects on the precipitation behavior of Nb, Ti, and V across different studies.

Based on this understanding, inclusion–precipitation coupling can be summarized at three mutually coupled levels: first, inclusion inheritance and interfacial reconstruction; second, local solute redistribution and altered precipitation sensitivity; and third, dynamic selection of the precipitation window through thermomechanical pathways [75,76,77,78].

First, rare-earth elements alter the speciation of inclusions retained after solidification through deoxidation, desulfurization, and inclusion modification. Rare-earth oxides, oxysulfides, and composite inclusions not only change the inclusion type but also reorganize crystallographic matching and interfacial energy states at the inclusion/matrix interface. Previous studies have shown that certain stable inclusions can significantly lower the intragranular nucleation energy barrier and alter subsequent phase transformation or precipitation behavior [79,80,81,82,83,84,85,86,87,88]. For microalloyed steels, not all rare-earth inclusions can serve as effective nucleation sites for NbC, TiN, (Nb,Ti)(C,N), or V(C,N). What truly matters is whether the inclusions possess low interfacial energy and suitable geometric and crystallographic conditions [89,90]. Therefore, the influence of rare-earth elements on precipitation behavior stems primarily from changes in the interfacial state, rather than merely from changes in the number of inclusions [91,92,93,94,95].

Second, the inclusion interface serves not only as a geometric carrier but also as a region in which the local solute field is restructured. Previous studies have shown that stable oxides can induce subsequent interfacial re-precipitation of TiN, MnS, and other compounds, leading to localized Mn depletion in adjacent regions and thereby increasing the local driving force for phase transformation [96]. This indicates that the role of inclusions is not limited to providing nucleation sites, but also includes modifying the local chemical environment through interfacial re-precipitation and elemental redistribution [97,98,99,100]. For microalloyed steels, this mechanism is also applicable to the precipitation of Nb, Ti, and V carbonitrides. When rare-earth elements alter the composition and interfacial energy of the inclusion interface, the local diffusion and enrichment behavior of C, N, Nb, Ti, and V also changes, thereby influencing the onset time, location, and sensitivity of precipitation [101,102,103,104,105,106,107].

**Figure 21:**
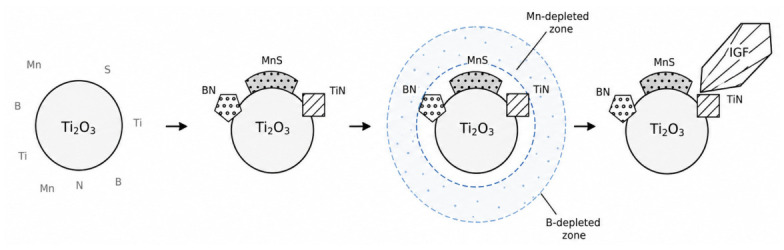
Schematic illustration of intragranular ferrite nucleation promoted by re-precipitation and local Mn depletion around stable oxide particles during cooling.

As shown in Figure 21, stable inclusions can enhance the local nucleation driving force through a combination of interfacial re-precipitation and local elemental depletion. Although this mechanism was initially proposed to explain the formation of intragranular ferrite, its broader theoretical significance lies in revealing a continuous process involving stable inclusions, interfacial re-precipitation, modification of the local chemical environment, and enhancement of the nucleation driving force. This concept is also applicable to understanding how rare-earth inclusions regulate the location and timing of Nb, Ti, and V carbonitride precipitation [108,109,110,111,112,113,114].

Furthermore, the potential precipitation conditions created by inclusions and interfaces can only become effective when the material enters the corresponding thermomechanical regime. Existing precipitation models indicate strong coupling between Nb/V carbonitride precipitation and austenite recrystallization. Once strain-induced precipitation is initiated, it significantly inhibits static recrystallization and gives rise to typical RPTT/PTT behavior [115,116,117,118,119,120]. When generalized to continuous cooling conditions, it becomes evident that precipitation behavior is controlled not only by the inclusion state, but also by rolling temperature, strain, cooling rate, and reheating regime [121,122,123,124]. Therefore, the inclusion state determines where precipitation is more likely to occur, whereas the thermomechanical path determines whether precipitation can occur and in what manner.

**Figure 22:**
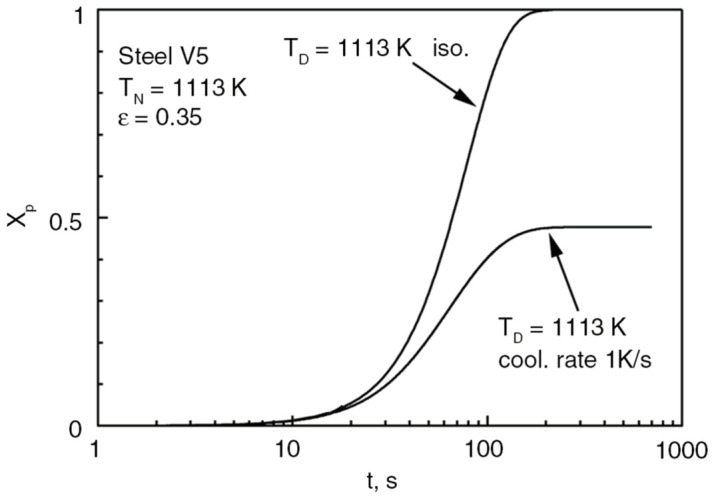
Comparison of precipitated fraction under isothermal and continuous cooling conditions in microalloyed steels [67].

Figure 22 shows that precipitation kinetics are highly sensitive to the thermal path. Even when the inclusion state remains the same, different rolling temperatures, cooling rates, or strain levels can lead to markedly different Nb/Ti/V precipitation responses. This is also one of the key reasons for the long-standing inconsistency in the literature regarding whether rare-earth elements promote or inhibit precipitation.

Consequently, this review proposes that, in microalloyed steels, trace rare-earth elements first modify the inclusion spectrum inherited after solidification through inclusion modification. Subsequently, these inclusions further reconfigure the interfacial energy, local stress field, and local solute field at the inclusion/matrix interface through changes in core–shell structure, interfacial composition, and size distribution. On this basis, the effective nucleation barriers, local compositional supersaturation, and diffusion fluxes of Nb, Ti, and V carbonitrides are altered. Ultimately, under specific heat-treatment and cooling profiles, these changes manifest as systematic shifts in precipitation onset time, peak precipitation temperature range, particle size, number density, and spatial distribution. The macroscopically observed strengthening, toughening, heat-affected-zone stabilization, fatigue behavior, and corrosion behavior can all be regarded as different external manifestations of inclusion–precipitation coupling.

This framework also explains why trace rare-earth elements can act as hidden metallurgical variables. The key does not lie in the average concentration itself, but in whether rare earths drive the system across certain interface-controlled thresholds. For example, they may shift originally ineffective inclusions into an effective nucleation size range, transform high-energy interfaces into low-energy interfaces, or synchronize a precipitation window that was previously mismatched with the hot-working process with the actual rolling and cooling path. Because these processes exhibit pronounced nonlinear characteristics, trace rare-earth elements display typical features of low addition levels and high metallurgical responsiveness.

Therefore, future research on trace rare-earth elements should not merely report total rare-earth content or inclusion types, but should further focus on the coupling relationships among inclusion lineage, hierarchical interfacial structures, local elemental segregation, and thermal processing paths. Only within such a framework can different research results be made truly comparable.

## 7. Conclusions

This review systematically examines the effects of trace and residual rare-earth elements on inclusion evolution and the precipitation behavior of Nb-, Ti-, and V-bearing microalloy precipitates in microalloyed steels, with particular emphasis on the coupling relationship among inclusion inheritance, interfacial states, and precipitation kinetics. The main conclusions are as follows:

(1) Trace rare-earth elements alter not only the final morphology of inclusions, but also their evolution pathways and inheritance mechanisms during solidification and cooling. With the addition of La or Ce, inclusions in steel can gradually transform from conventional types such as Al_2_O_3_ and MnS into rare-earth oxides and oxysulfides. This transformation further changes the size, interfacial structure, and thermal stability of inclusions, thereby reshaping the foundation for subsequent microstructural evolution.
(2) The influence of rare-earth elements on the precipitation behavior of Nb-, Ti-, and V-bearing carbonitrides does not follow a simple and universal pattern of promotion or inhibition. Rare-earth-modified inclusions may act as effective heterogeneous nucleation sites and promote precipitation, or they may alter the precipitation location, timing, and coarsening behavior through interfacial segregation, interfacial energy restructuring, and local solute redistribution. Therefore, the discrepancies among reported results in the literature essentially arise from differences in interfacial states, local solute environments, and thermomechanical pathways.
(3) Based on existing research, trace rare-earth elements first alter the inherited inclusion lineage, then reconfigure the interfacial energy and local solute field at the inclusion/matrix interface, and ultimately lead to systematic changes in the precipitation behavior and microstructural properties of Nb-, Ti-, and V-microalloyed steels under specific heat-treatment and cooling conditions. Future research should move beyond inclusion modification alone and further consider the regulation of interfacial states and precipitation behavior, while strengthening the integration of in situ characterization and multiscale simulation.
(4) A key requirement for future research is to establish direct correlations among inclusion chemistry, interfacial structure, local rare-earth segregation, and precipitation kinetics. This requires integrated characterization approaches combining total rare-earth analysis, inclusion extraction, automated SEM–EDS inclusion statistics, high-resolution TEM/STEM–EDS or APT interfacial characterization, and precipitation-kinetics measurements such as SANS, electrical resistivity, or in situ thermomechanical characterization. Such correlated evidence is necessary to clarify whether rare earths act mainly through inclusion-controlled nucleation, solute-state effects, segregation-controlled interfacial regulation, or their combined action.

## Figures and Tables

**Table 1:** Occurrence states of trace and residual rare earth in steels and their metallurgical implications.

Steel System	Main Occurrence State of Rare Earth	Main Action Site	Key Implication	Ref.
Experimental steels with different T.RE.	RE inclusions + solid-solution RE	Matrix and inclusions	Total rare earth content does not directly represent effective rare earth content; effective rare earth content is significantly influenced by total rare earth content, O, S, Al, and thermal history	[10,13]
High-strength wheel steels	RE oxides/sulfides, solid-solution Ce, Ce-rich nanoclusters, Ce–S–As/Ce–S–As–P inclusions	Inclusions, grain boundaries, local defect regions	The evolution of rare earth elements from a compound state to a solid solution and impurity-coupled state confirms that rare earth elements are dynamic state variables rather than simple compositional variables	[11]
TRIP steels	RE clusters and solid-solution RE	Ferrite matrix, dislocations, phase boundaries	Rare earth elements can directly contribute to local diffusion and the stabilization of carbide-related microstructures, rather than merely acting through modified inclusions	[12]
Fe melts containing As	Ce–S–As/CeAs-type inclusions	Grain boundaries and impurity-segregated regions	Residual rare earth elements can restore the local chemical environment by immobilizing harmful residual elements such as arsenic	[29]
SA508-III steel	Grain-boundary segregated Ce	Grain boundaries	Even at extremely low average concentrations, trace amounts of Ce can strengthen grain boundaries through segregation	[30]
SA508-4N steel	Segregated/dissolved Ce	Grain boundaries	Trace rare earth elements exhibit localized effects rather than effects of average distribution	[31]

**Table 2:** Reported lattice mismatch values for representative rare-earth inclusion/precipitate interfaces.

Rare-Earth-Related Substrate	Precipitate/Nucleating Phase	Reported Lattice Mismatch (%)	Nucleation Implication	Ref.
CeAlO_3_	TiN	7.55	Moderate lattice matching; effective heterogeneous nucleation site for TiN	[39]
Ce_2_O_2_S	TiN	7.90	Moderate lattice matching; effective heterogeneous nucleation site for TiN	[39]
Al_2_O_3_	TiN	10.91	Conventional oxide reference; lower nucleation potency than CeAlO_3_ and Ce_2_O_2_S	[39]
YAlO_3_	NbC	~5.4	Low mismatch; favorable heterogeneous nucleation substrate for NbC	[38]
Y_2_O_3_	VC	3.63	Very low mismatch; strong nucleation potency for VC	[46]
Y_2_O_3_	NbC	6.8	Moderate lattice matching; potential heterogeneous nucleation substrate for NbC	[48]

**Table 3:** Comparison of Mechanisms for the Promotion and Inhibition of Microalloying Precipitation Phases by Trace/Residual Rare Earth Elements.

Dominant Scenario	Rare-Earth-Related Target	Observed Response	Underlying Mechanism	Ref.
Promotion of fine TiN/NbC/VC precipitation	Fine CeAlO_3_, Ce_2_O_2_S, YAlO_3_, and Y_2_O_3_ particles	Increased number density, reduced particle size, and more dispersed distribution	Low lattice mismatch heterogeneous nucleation and reduced interfacial energy	[38,39,46,48]
Promotion of coarse composite precipitates	Coarse Ce-rich clustered cores	Formation of TiN clusters or large composite precipitates	Although nucleation sites increase, clustered cores promote encapsulation-type coarsening	[39,45]
Suppression of multilayer carbonitride precipitation	High-mismatch Ce-O and Ce-O-S particles	Reduced multilayer (Ti,V)(C,N) structures and fewer coarse precipitates	Original heterogeneous nucleation pathways are disrupted	[43]
Initial promotion followed by suppression	Ce_2_O_3_/Ce_2_O_2_S particles combined with dendrite refinement	Area fraction of primary carbides first increases and then decreases	Competition between heterogeneous nucleation promotion and microstructure-refinement-induced suppression	[44]

**Table 4:** Major mechanisms by which trace/residual rare earths regulate microalloy precipitates through inclusions.

Rare-Earth-Related Inclusion/State	Main Associated Precipitate	Primary Role	Resulting Effect	Ref.
CeAlO_3_, Ce_2_O_2_S	TiN	Heterogeneous nucleation core	Reduced TiN nucleation barrier, increased number density, and refined particle size	[39,40]
YAlO_3_	TiN, NbC	YAlO_3_ first serves as a heterogeneous nucleation substrate for TiN, which subsequently provides nucleation sites for NbC precipitation	Formation of multilayer structures such as YAlO_3_-TiN/(Ti,V)N-NbC/(Nb,Mo)C	[42]
YAlO_3_	NbC	Low-misfit stable interface	Low lattice mismatch and interfacial energy at the YAlO_3_/NbC interface enable direct NbC nucleation	[38]
Y_2_O_3_	VC, Cr_23_C_6_	Coupled heterogeneous nucleation and interfacial diffusion	Provides nucleation sites while promoting interfacial interdiffusion and stacking-fault structures	[46]
Ce–O, Ce–O–S	(Ti,V)(C,N), etc.	Suppression of heterogeneous nucleation	Rare-earth oxides/oxysulfides do not always promote precipitation; some suppress multilayer carbonitride formation	[43]
Dissolved/segregated Ce and Y	Laves phase, M_23_C_6_, etc.	Modification of interfacial segregation and precipitation sensitivity	Rearrangement of precipitation behavior through grain-boundary site competition, selective solute attraction, and altered local diffusion pathways	[49,50]

**Table 5:** Effects of rare-earth elements on precipitation behavior in microalloyed steels and related alloy systems.

Steel Grade	RE	RE Content (wt.%)	Inclusion/Nucleation Site	Precipitate Phase	Reported Observation	Dominant Kinetic Stage Affected	Proposed Mechanism	Ref.
Nb-microalloyed steel	La	0.0048	—	NbC	Strain-induced NbC precipitation in austenite was delayed	Dissolution/solubility control; nucleation delay	Increased solubility of Nb and C in γ-Fe	[6]
Nb-microalloyed steel	La	0.0048	—	NbC	NbC precipitation in ferrite was accelerated	Nucleation and early growth acceleration	Reduced solubility of Nb and C in α-Fe	[7]
Ultra-high-strength steel	Ce	0.009	—	Ti(C,N)	Area fraction increased and particle size decreased	Nucleation enhancement and growth refinement	Enhanced precipitation tendency and refinement	[45]
High-Ti steel	Ce	0.0015–0.0165	CeAlO_3_, Ce_2_O_2_S	TiN	Number density increased from 38.6 to 105.8 mm^−2^	Heterogeneous nucleation; possible coarsening at high Ce	Heterogeneous nucleation on RE inclusions	[39]
H13 steel	Y	0.013	Y_2_O_3_	VC	VC preferentially precipitated on Y_2_O_3_ particles	Heterogeneous nucleation and interface-controlled growth	Low lattice mismatch and interfacial nucleation	[34]
H13 steel	Y	0.013	Y_2_O_3_	Cr_23_C_6_	Attached precipitation observed	Interface-controlled nucleation and growth	Coupled interfacial diffusion and heterogeneous nucleation	[34]
11Cr ferritic/martensitic steel	Y	0.10	YAlO_3_	TiN → NbC/(Nb,Mo)C	Hierarchical multilayer precipitation structure formed	Sequential nucleation and shell growth	Sequential heterogeneous nucleation	[33]
Fe-Cr-C alloy	Y	—	YAlO_3_	NbC	Direct nucleation of NbC on YAlO_3_	Heterogeneous nucleation	Low lattice mismatch (~5.4%)	[32]
316LN stainless steel	Ce	0.032	TiN	Laves phase	Fine Laves phase precipitation increased	Nucleation enhancement and pathway selection	Heterogeneous nucleation and phase-selection effect	[47]
316LN stainless steel	Ce	0.032	—	σ phase	Sigma-phase precipitation suppressed	Pathway switching/suppression of competing phase	Modification of precipitation pathway	[47]

**Table 6:** Major origins of contradictory precipitation responses and their metallurgical nature.

Apparent Contradiction	Hidden State Variable	Metallurgical Nature	Ref.
In the same microalloyed system, some studies report that deformation strongly promotes precipitation, whereas others suggest only limited promotion	Whether unrecrystallized austenite is retained after deformation; retention of dislocation density and substructures	Deformation not only increases nucleation-site density, but also modifies growth/coarsening pathways through pipe diffusion along dislocations; once recrystallization occurs first, precipitation is markedly delayed	[51,52,56,59]
Thermodynamic calculations predict precipitation, but experiments still show weak or undetectable precipitation after prolonged holding	Kinetic accessibility; diffusion pathways; whether phase transformation has initiated	Equilibrium phase diagrams only provide thermodynamic limits and do not guarantee precipitation within finite holding times; high-temperature regions are often “thermodynamically allowed but kinetically sluggish”	[58,60]
High V/C contents are sometimes considered to promote precipitation, but in other studies are reported to delay precipitation	Final precipitation amount and precipitation-start timing are not equivalent indicators	Increased V/C can raise the final precipitate fraction or number density, while simultaneously delaying the γ→α transformation and thus postponing the observable onset of precipitation	[58,60,61]
Different studies on the same steel grade report different precipitate types and compositions	Different fractions of dissolved solute after reheating; whether undissolved TiN/(Ti,Nb)(C,N) particles remain	Undissolved particles act both as solute sinks and heterogeneous nucleation substrates, thereby altering subsequent precipitation composition and spatial distribution	[53,55,57,62]
Some studies consider interphase precipitation most beneficial, whereas others emphasize the importance of random precipitation	Different precipitation mechanisms; different evaluation criteria	Interphase precipitation and random precipitation differ fundamentally in number density, size distribution, preferential location, and strengthening mechanism, and therefore cannot be evaluated using a single metric	[63]

**Table 7:** Information range and limitations of different characterization methods for precipitation responses.

Method	Information to Which the Method Is Most Sensitive	Main Advantage	Main Limitation	Ref.
Stress relaxation/softening analysis	Precipitation-start time; recrystallization–precipitation coupling	Effective for capturing the kinetic onset of strain-induced precipitation	Cannot directly provide the actual size distribution or chemical composition of precipitates	[51,52,56,58]
Electrical resistivity measurement	Solute depletion and precipitation initiation/progression	Sensitive to precipitation onset and suitable for constructing PTT curves quantitatively	Cannot directly distinguish different nucleation sites or precipitate types	[52,58,61]
TEM/carbon extraction replica	Nucleation sites, morphology, orientation relationships, and local particle size	Enables direct observation of grain-boundary, dislocation, and interphase precipitates	Limited statistical volume; constrained resolution for extremely fine or early-stage precipitates	[53,54,55,56,57,58,63]
Atom probe tomography (APT)	Atomic-scale compositional gradients and core–shell evolution	Most suitable for revealing stoichiometry and elemental segregation	Small sampling volume and insufficient for overall statistical representation	[59]
Small-angle neutron scattering (SANS)	Average radius, volume fraction, number density, and size distribution	Large statistical sampling volume and suitable for precipitation kinetics quantification	Requires TEM/APT support for shape and composition assumptions	[59,60,63]
Thermodynamic equilibrium calculation	Equilibrium phases, solubility, and theoretical upper limit of precipitate fraction	Useful for identifying potential precipitation regions and equilibrium limits	Does not account for actual kinetic retardation or defect-related effects	[36,59,60]

## Data Availability

No new data were created or analyzed in this study. Data sharing is not applicable to this article.
